# Supplementary material for: The association of SARS-CoV-2 infection and tuberculosis disease with unfavorable treatment outcomes: A systematic review
Source: PLOS Glob Public Health. 2023 Jul 19;3(7):e0002163. doi: 10.1371/journal.pgph.0002163 (PMC10355446; doi:10.1371/journal.pgph.0002163)
Supplement: S1 Text — Table A in S1 Text. Search Strategies for Different Databases. Table B in S1 Text. PECO Question: In people with tuberculosis disease, does SARS-CoV-2 infection influence outcomes? Table C in S1 Text. Adapted Newcastle-Ottawa Scale for Quality Assessment for Cohort and Case-Control Studies. Table D in S1 Text. List of Excluded Studies (Non-Duplicate) at Full-Text Stage and Reasons for Not Sharing Data. Table E in S1 Text. Mortality Among Select Populations in Each Study Evaluating TB Patients Co-infected with SARS-CoV-2 vs. TB Patients without SARS-CoV-2. Table F in S1 Text. Loss to Follow-up Among Select Populations in Each Study Evaluating TB Patients Co-infected with SARS-CoV-2 vs. TB Patients without SARS-CoV-2. Table G in S1 Text. Treatment Failure Among Select Populations in Each Study Evaluating TB Patients Co-infected with SARS-CoV-2 vs. TB Patients without SARS-CoV-2. Table H in S1 Text. Evidence Profile. (DOCX) [file pgph.0002163.s001.docx]

**S1 Text**

Contents

[Table A. Search Strategies for Different Databases 2](#_Toc138700928)

[Table B. PECO Question: In people with tuberculosis disease, does SARS-CoV-2 infection influence outcomes? 5](#_Toc138700929)

[Table C. Adapted Newcastle-Ottawa Scale for Quality Assessment for Cohort and Case-Control Studies 6](#_Toc138700930)

[Table D. List of Excluded Studies (Non-Duplicate) at Full-Text Stage and Reasons for Not Sharing Data 7](#_Toc138700931)

[Table E. Mortality Among Select Populations in Each Study Evaluating TB Patients Co-infected with SARS-CoV-2 vs. TB Patients without SARS-CoV-2 9](#_Toc138700932)

[Table F. Loss to Follow-up Among Select Populations in Each Study Evaluating TB Patients Co-infected with SARS-CoV-2 vs. TB Patients without SARS-CoV-2 10](#_Toc138700933)

[Table G. Treatment Failure Among Select Populations in Each Study Evaluating TB Patients Co-infected with SARS-CoV-2 vs. TB Patients without SARS-CoV-2 11](#_Toc138700934)

[Table H. Evidence Profile 12](#_Toc138700935)

# Table A. Search Strategies for Different Databases

| **Ovid MEDLINE and Epub Ahead of Print, In-Process, In-Data-Review & Other Non-Indexed Citations, Daily and Versions**  **1946 to February 4, 2022** | | |
| --- | --- | --- |
| **Search Number** | **Search Terms** | **Records Identified** |
| 1 | sars-related coronavirus/ | 4012 |
| 2 | (coronavirinae/ or betacoronavirus/ or coronavirus infection/) and (epidemic/ or pandemic/) | 39,230 |
| 3 | (nCoV* or 2019nCoV or 19nCoV or COVID19* or COVID or SARS-COV-2 or SARSCOV-2 or SARS-COV2 or SARSCOV2 or SARS coronavirus 2 or Severe Acute Respiratory Syndrome Coronavirus 2 or Severe Acute Respiratory Syndrome Corona Virus 2).ti,ab,kw,hw,ot. | 221,787 |
| 4 | (longCOVID* or postCOVID* or postcoronavirus* or postSARS*).ti,ab,kw,hw,ot. | 26 |
| 5 | ((coronavirus* or corona virus* or betacoronavirus*) adj3 (pandemic* or epidemic* or outbreak* or crisis)).ti,ab,kw,ot. | 13,397 |
| 6 | ((Wuhan or Hubei) adj5 pneumonia).ti,ab,kw,ot. | 379 |
| 7 | ((new or novel or “19” or “2019” or Wuhan or Hubei or China or Chinese) adj3 (coronavirus* or corona virus* or betacoronavirus* or CoV or HcoV)).ti,ab,kw,hw,ot. | 61,091 |
| 8 | or/1-7 | 227,532 |
| 9 | (cov2 or “sars 2” or “coronavirus 2” or ncp).ti,ab,kf. | 27,545 |
| 10 | exp tuberculosis/ | 200,254 |
| 11 | (tuberculosis or mycobacterium tuberculosis or TB or MDRTB or XDRTB or ATB or active tuberculosis or RRTB or Mtb).tw,kf. | 250,324 |
| 12 | 8 or 9 | 229,208 |
| 13 | 10 or 11 | 285,659 |
| 14 | 12 and 13 | 1220 |
| 15 | limit 14 to (human and yr=”2020 -Current”) | 861 |

| **Embase**  **1974 to February 3, 2022** | | |
| --- | --- | --- |
| **Search Number** | **Search Terms** | **Records Identified** |
| 1 | sars-related coronavirus/ | 487 |
| 2 | (coronavirinae/ or betacoronavirus/ or coronavirus infection/) and (epidemic/ or pandemic/) | 10,719 |
| 3 | (nCoV* or 2019nCoV or 19nCoV or COVID19* or COVID or SARS-COV-2 or SARSCOV-2 or SARS-COV2 or SARSCOV2 or SARS coronavirus 2 or Severe Acute Respiratory Syndrome Coronavirus 2 or Severe Acute Respiratory Syndrome Corona Virus 2).ti,ab,kw,hw,ot. | 224,227 |
| 4 | (longCOVID* or postCOVID* or postcoronavirus* or postSARS*).ti,ab,kw,hw,ot. | 72 |
| 5 | ((coronavirus* or corona virus* or betacoronavirus*) adj3 (pandemic* or epidemic* or outbreak* or crisis)).ti,ab,kw,ot. | 12,993 |
| 6 | ((Wuhan or Hubei) adj5 pneumonia).ti,ab,kw,ot. | 433 |
| 7 | ((new or novel or “19” or “2019” or Wuhan or Hubei or China or Chinese) adj3 (coronavirus* or corona virus* or betacoronavirus* or CoV or HcoV)).ti,ab,kw,hw,ot. | 195,452 |
| 8 | (cov2 or “sars 2” or “coronavirus 2” or ncp).ti,ab,kf. | 28,087 |
| 9 | or/1-8 | 245,534 |
| 10 | exp tuberculosis/ | 205,572 |
| 11 | (tuberculosis or mycobacterium tuberculosis or TB or MDRTB or XDRTB or ATB or active tuberculosis or RRTB or Mtb).tw,kf. | 226,148 |
| 12 | or/10-11 | 279,290 |
| 13 | 9 and 12 | 2156 |
| 14 | limit 13 to (human and yr=”2020 -Current”) | 2055 |

**WHO Library Database Search Strategy—Completed February 6, 2022**

Website: <https://search.bvsalud.org/global-literature-on-novel-coronavirus-2019-ncov/>

Timeframe: 2019 to February 6, 2022

Search terms: “Tuberculos*” under “Title, abstract, subject”

Results: 2352 records

**SCOPUS Database Search Strategy—Completed February 6, 2022**

Timeframe: 2020 to February 6, 2022

Search terms: (TITLE-ABS-KEY (**covid** OR **sars-cov-2**) AND TITLE-ABS-KEY (**tuberculosis** OR **mycobacterium** AND **tuberculosis** OR **tb**)) AND PUBYEAR > 2018

Results: 1719 records

**Web of Science Database Search Strategy—Completed February 6, 2022**

Timeframe: 2020 to February 6, 2022

Search terms: (nCoV* or 2019nCoV or 19nCoV or COVID19* or COVID or SARS-COV-2 or SARSCOV-2 or SARS-COV2 or SARSCOV2 or SARS coronavirus 2 or Severe Acute Respiratory Syndrome Coronavirus 2 or Severe Acute Respiratory Syndrome Corona Virus 2) and (tuberculosis or mycobacterium tuberculosis or TB or mdrtb or xdrtb or ATB or active tuberculosis or rrtb or Mtb)

Results: 2480 records

**MedRxiv and BioRxiv Search Strategy—Completed February 5, 2022**

We used the medrxivr package (<https://cran.r-project.org/web/packages/medrxivr/index.html>) in R to execute the search from March 1, 2020, to February 5, 2022.

We first identified all records that had any version of the words “covid” or “sars” in the title and/or abstract. From here, we narrowed the identified records to only records that mentioned any version of the word “tuberculosis” or the specific word “TB” in the title and/or abstract. We then subset the identified records on only the most recently posted preprint.

Results: 62 preprints

# Table B. PECO Question: In people with tuberculosis disease, does SARS-CoV-2 infection influence outcomes?

| **Population** | **Exposure** | **Comparator** | **Outcome** |
| --- | --- | --- | --- |
| People of any age diagnosed with tuberculosis disease that is microbiologically confirmed (primary population) and/or clinically diagnosed (secondary population).  Subgroups of interest   - Age (≥60y or other definition of elderly; <5y; <19y or other definition of child and adolescent) - Biological sex (male; female) - Pregnancy - Physical co-morbidities (diabetes mellitus; HIV; obesity; undernutrition; COPD; cardiovascular disease; cancer; chronic kidney disease; physical disability) - Mental co-morbidities (mood and anxiety disorders; psychotic disorders) - Health behaviors (smoking; alcohol use disorder; substance use disorder) - Tuberculosis type (pulmonary; extrapulmonary; meningitis; miliary) - Drug-resistance (drug-sensitive; drug-resistant; isoniazid-resistant; rifampicin-resistant) - Clinical Findings (sputum smear positive; lung cavitation; “severe” disease, as defined by authors) - Microbiologic confirmation (culture, smear, or nucleic acid amplification test; clinically diagnosed) - Receipt of appropriate tuberculosis treatment - Tuberculosis treatment delay from diagnosis (<14 days; ≥14 days) - Location of treatment (ambulatory; hospital; intensive care unit) - SARS-CoV-2 vaccination status (type; number of doses; time since last dose) - SARS-CoV-2 severity - SARS-CoV-2 variant (wild-type; alpha; beta; gamma; delta; omicron) - SARS-CoV-2 therapeutic use - SARS-CoV-2 confirmation (confirmed; probable or suspected; antibody or previous positive; self-report) | There are two exposures of interest within the population (the corresponding list number in exposure is compared to the same list number in comparator, i.e., (1) vs. (1) and (2) vs. (2)):   1. Confirmed (primary population) SARS-CoV-2 infection as per WHO definition and/or suspected or possible (secondary population) SARS-CoV-2 infection as per WHO definition. If possible, stratified by difference in time from tuberculosis treatment and SARS-CoV-2 diagnosis (<6 months to ≥28 days before; <28 days before to <28 days after; ≥28 days after to treatment discontinuation). 2. Confirmed (primary population) SARS-CoV-2 infection as per WHO definition and/or suspected or possible (secondary population) SARS-CoV-2 infection as per WHO definition and receiving steroids or other immunomodulating treatment for SARS-CoV-2. If possible, stratified by difference in time from tuberculosis treatment and SARS-CoV-2 diagnosis (<6 months to ≥28 days before; <28 days before to <28 days after; ≥28 days after to treatment discontinuation). | There are two exposures of interest within the population (the corresponding list number in comparator is compared to the same list number in exposure, i.e., (1) vs. (1) and (2) vs. (2)):   1. No SARS-CoV-2 infection 2. Confirmed (primary population) SARS-CoV-2 infection as per WHO definition and/or suspected or possible (secondary population) SARS-CoV-2 infection as per WHO definition and **NOT** receiving steroids or other immunomodulating treatment for SARS-CoV-2. If possible, stratified by difference in time from tuberculosis treatment and SARS-CoV-2 diagnosis (<6 months to ≥28 days before; <28 days before to <28 days after; ≥28 days after to treatment discontinuation). | The primary outcome of interest for both comparisons is unsuccessful tuberculosis treatment outcome (death, treatment failure or recurrence, loss to follow-up). For comparison (2), the co-primary outcome of interest is progression of SARS-CoV-2 (severe disease, admission to intensive care unit, need for mechanical ventilation, death).  Secondary outcomes of interest for both comparisons:   - All-cause mortality - Loss to follow-up - Failure or recurrence - Microbiologic conversion by end of 2 months of treatment or time-to-conversion - Hospitalization during treatment - Sequelae, disability, other quality-of-life indices and patient-important outcomes - Total duration of tuberculosis treatment - Duration of hospitalization - Time to symptom resolution - Medication adherence (proportion of doses taken)   Secondary outcomes of interest for only comparison 2:   - Need for oxygen supplementation - Need for non-invasive or invasive ventilation - Need for vasopressors - Need for anticoagulants - Need for renal replacement therapy - Presence of symptoms consistent with “long COVID” |

# Table C. Adapted Newcastle-Ottawa Scale for Quality Assessment for Cohort and Case-Control Studies

| **Question** | **Response** | **Score** |
| --- | --- | --- |
| **DOMAIN 1: RISK OF BIAS RELATED TO SELECTION AND GROUP ASSIGNMENT** | | |
| 1. COHORT: Is the “intervention/exposure” cohort truly or somewhat representative of the average TB patient? 2. CASE-CONTROL: Is there minimal selection bias from the selected cases (e.g., consecutive cases or obviously representative) | Yes  No  Unclear | 1  0  0 |
| 1. COHORT: Is the selection of the “control/comparator” cohort from the same community as the “intervention” cohort? 2. CASE-CONTROL: Is the selection of the “control” cohort from the community or a similar population as the cases? | Yes  No  Unclear | 1  0  0 |
| 1. COHORT: Is there low risk of bias for the ascertainment of the diagnosis/exposure in the intervention/exposure group (i.e., CONFIRMED for both TB and SARS-CoV-2)? 2. CASE-CONTROL: Is the definition of a case adequate, with independent validation (i.e., CONFIRMED for both TB and SARS-CoV-2)? | Yes  No  Unclear | 1  0  0 |
| 1. COHORT: For both the “intervention”/”exposure” and the comparator groups, there is no risk that the outcome of interest was present at the start of the study. 2. CASE-CONTROL: For the controls, there is no risk that they have history or presence of the outcome of interest. | Yes  No  Unclear | 1  0  0 |
| **DOMAIN 2: RISK OF BIAS RELATED TO COMPARABILITY OF POPULATIONS** | | |
| 1. COHORT & CASE-CONTROL: The study attempts to control for and address important factors that would impact the comparability of the intervention/exposed and the comparator groups **OR** the study populations are highly comparable at baseline. | Yes  No  Unclear | 1  0  0 |
| 1. COHORT & CASE-CONTROL: Was the context, management, and processes for care for participants in the intervention/exposure group and the comparator group highly comparable except for the intervention/exposure? | Yes  No  Unclear | 1  0  0 |
| **DOMAIN 3: RISK OF BIAS RELATED TO OUTCOME ASSESSMENT AND/OR ASCERTAINMENT** | | |
| 1. COHORT & CASE CONTROL: Was the assessment of the outcome rigorously collected and evaluated (e.g., independent blind assessment, record linkage, met case definition, structured interview) | Yes  No  Unclear | 1  0  0 |
| 1. COHORT: Was the follow-up of the study long enough for outcomes to occur (i.e., to treatment completion)? 2. CASE-CONTROL: Were outcomes assessed and obtained in identical fashions for both exposed/intervention and comparator groups? | Yes  No  Unclear | 1  0  0 |
| 1. COHORT: Was there a low risk of bias associated with those lost to follow-up (i.e., <10% of cohort lost and description of when people dropped out)? 2. CASE-CONTROL: Was the participation/response rate similar for both cases and controls? | Yes  No  Unclear | 1  0  0 |

*Note: a score of 7 or higher with questions 2, 5, and 7, with “yes” responses were considered high quality.

# Table D. List of Excluded Studies (Non-Duplicate) at Full-Text Stage and Reasons for Not Sharing Data

| **Author** | **Year** | **Title** | **Journal** | **Author Contacted** | **Reason for Exclusion** | **Notes** |
| --- | --- | --- | --- | --- | --- | --- |
| Agrupis | 2021 | Epidemiological and clinical characteristics of the first 500 confirmed COVID-19 inpatients in a tertiary infectious disease referral hospital in Manila, Philippines | Tropical Medicine and Health | No | No relevant data in report | Report did not suggest authors may have relevant data |
| Anuradha | 2021 | Co-infection of COVID-19 and tuberculosis in a tertiary care hospital | Journal of Clinical & Diagnostic Research | Yes | No relevant data in report | Author replied that they did not have relevant data |
| Arghir | 2022 | Severe COVID-19 Associated with Advanced Forms of Pulmonary Tuberculosis Disease in an Endemic Area of Romania | Chest | No | Conference Abstract | N/A |
| Bandara | 2021 | The cruel dual; SARS-CoV-2 and tuberculosis co-infection, the Sri Lankan experience | Respirology | No | Conference Abstract | N/A |
| Demkina | 2020 | Risk factors for outcomes of COVID-19 patients: an observational study of 795 572 patients in Russia | medRxiv | No | No relevant data in report | Report did not suggest authors may have relevant data |
| Gajbhiye | 2021 | Clinical characteristics, outcomes, & mortality in pregnant women with COVID-19 in Maharashtra, India: Results from PregCovid registry | Indian Journal of Medical Research | Yes | No relevant data in report | Author did not respond to our attempts to contact them |
| Gupta | 2020 | A profile of a retrospective cohort of 22 patients with COVID-19 and active/treated tuberculosis | European Respiratory Journal | Yes | Cohort Overlaps with Another Included Study | N/A |
| Gupta | 2020 | Impact of COVID-19 on tuberculosis services in India | International Journal of Tuberculosis and Lung Disease | No | Incorrect Study Design | N/A |
| Lagrutta | 2021 | The Febrile Emergency Unit at Muniz Hospital facing COVID-19, HIV and tuberculosis | Medicina (Buenos Aires) | Yes | No relevant data in report | Author did not share relevant data after initial contact |
| Liu | 2020 | Active or latent tuberculosis increases susceptibility to COVID-19 and disease severity | medRxiv | No | <10 Tuberculosis Patients Included | N/A |
| Malashenkov | 2021 | Novel coronavirus infection (CoVID-19) in tuberculosis patients in St. Petersburg | Jurnal Infektologii | Yes | No relevant data in report | Author did not respond to our attempts to contact them |
| Mash | 2021 | Evaluation of patient characteristics, management and outcomes for COVID-19 at district hospitals in the Western Cape, South Africa: Descriptive observational study | BMJ Open | Yes | No relevant data in report | Author did not respond to our attempts to contact them |
| Mishra | 2021 | Review of clinical profile, risk factors, and outcomes in patients with tuberculosis and covid-19 | Acta Biomedica | No | Incorrect Study Design | N/A |
| Motta | 2020 | Tuberculosis, COVID-19 and migrants: Preliminary analysis of deaths occurring in 69 patients from two cohorts | Pulmonology | No | Cohort Overlaps with Another Included Study | N/A |
| Mutyambizi | 2021 | Effect of COVID-19 on HIV, tuberculosis, and prevention of mother-to-child transmission of HIV indicators in Mopani district, South Africa | The South African Medical Journal | No | Incorrect Study Design | N/A |
| Nabity | 2021 | Sociodemographic Characteristics, Comorbidities, and Mortality among Persons Diagnosed with Tuberculosis and COVID-19 in Close Succession in California, 2020 | JAMA Network Open | Yes | No relevant data in report | Author replied that they did not have relevant data |
| Oh | 2021 | Impact of coronavirus disease-2019 on chronic respiratory disease in South Korea: an NHIS COVID-19 database cohort study | BMC Pulmonary Medicine | No | Incorrect Study Design | N/A |
| Orooj | 2021 | Impact of COVID-19 pandemic on quality of life and medication adherence among pulmonary Tb patients | International Journal of Current Research and Review | No | Incorrect Study Design | N/A |
| Patel | 2020 | A retrospective study of COVID-19 disease in confirmed tuberculosis patients from a tertiary care centre in Ahmedabad | National Journal of Community Medicine | No | No relevant data in report | Report did not suggest authors may have relevant data |
| Radulescu | 2021 | Tuberculosis and COVID-19 co-infection – clinical characteristics | Acta Medica Transilvanica | Yes | No relevant data in report | Author did not respond to our attempts to contact them |
| Rishad | 2021 | Variable presentations of tuberculosis during COVID-19 pandemic-a case series | Journal of Medicine | No | <10 Tuberculosis Patients Included | N/A |
| Sharif | 2021 | Prevalence and impact of comorbidities on disease prognosis among patients with COVID-19 in Bangladesh: A nationwide study amid the second wave | Diabetes & Metabolic Syndrome: Clinical Research & Reviews | No | <10 Tuberculosis Patients Included | N/A |
| Sy | 2020 | Previous and active tuberculosis increases risk of death and prolongs recovery in patients with COVID-19 | Infectious Diseases (London) | Yes | No relevant data in report | Author replied that they did not have relevant data |
| Syahridha | 2021 | Associated factors of the results of pulmonary tuberculosis treatment during the covid-19 pandemic in Makassar city | Open Access Macedonian Journal of Medical Sciences | Yes | No relevant data in report | Author did not respond to our attempts to contact them |
| Tadolini | 2020 | Active tuberculosis, sequelae and COVID-19 co-infection: first cohort of 49 cases | European Respiratory Journal | No | Cohort Overlaps with Another Included Study | N/A |
| Tinoco | 2022 | Impact of COVID-19 on extrapulmonary TB and the benefit of decentralised TB services | International Journal of Tuberculosis and Lung Disease | No | Incorrect Study Design | N/A |
| Tuberculosis Research Centre, India | 2021 | Effect of SARS-CoV-2 Disease on Immune Responses, Disease Severity and Treatment Outcomes in Pulmonary Tuberculosis | ClinicalTrials.gov | No | Ongoing Clinical Trial | N/A |
| Vanzetti | 2020 | Tuberculosis and covid-19 coinfection | Medicina (Buenos Aires) | No | <10 Tuberculosis Patients Included | N/A |
| Wang | 2021 | Influence of COVID-19 in patients with concurrent tuberculosis coinfections | Journal of Medical Virology | No | Incorrect Study Design | N/A |
| Western Cape Department of Health in collaboration with the National Institute for Communicable Diseases, South Africa | 2021 | Risk Factors for Coronavirus Disease 2019 (COVID-19) Death in a Population Cohort Study from the Western Cape Province, South Africa | Clinical Infectious Diseases | Yes | No relevant data in report | Author provided us data from a more recent report that is relevant |
| World Health Organization | 2021 | Effects of COVID-19 on people with current or previous tuberculosis — based on information as of 9 December 2020 | Online WHO Report | No | Incorrect Study Design | N/A |

**Summary of five studies that did not share data:** Among the five authors who replied but did not share data, two did not collect data on corticosteroids or other immunomodulating treatments, one did not respond to the follow-up email requesting specific data, one did not have TB outcome data, and one study was nested within another study already contributing data.

# Table E. Mortality Among Select Populations in Each Study Evaluating TB Patients Co-infected with SARS-CoV-2 vs. TB Patients without SARS-CoV-2

| **Author**  **(Year)** | **Population Subgroup** | **Outcome** | **Participants with Known Outcome** | **Participants with Outcome (%)** | **Odds Ratio (95% CI) for Outcome if Co-Infected with SARS-CoV-2** |
| --- | --- | --- | --- | --- | --- |
| Kilic  (2022)^1^ | All Participants  (All SARS-CoV-2 diagnosed >28 days after TB) | All-Cause Mortality | TB and SARS-CoV-2 Co-Infected: 4  TB Only: 16 | TB and SARS-CoV-2 Co-Infected: 0 (0%)  TB Only: 0 (0%) | N/A |
| Mohr-Holland  (2021)^2^ | All Participants | All-Cause Mortality | TB and SARS-CoV-2 Co-Infected: 27  TB Only: 54 | TB and SARS-CoV-2 Co-Infected: 13 (48%)  TB Only: 17 (31%) | 2.02 (0.78 to 5.22) |
|  | Only SARS-CoV-2 diagnosed within 28 days of TB | All-Cause Mortality | TB and SARS-CoV-2 Co-Infected: 7  TB Only: 54 | TB and SARS-CoV-2 Co-Infected: 3 (43%)  TB Only: 17 (31%) | 1.63 (0.33 to 8.11) |
|  | Only SARS-CoV-2 diagnosed >28 days after TB | All-Cause Mortality | TB and SARS-CoV-2 Co-Infected: 20  TB Only: 54 | TB and SARS-CoV-2 Co-Infected: 10 (50%)  TB Only: 17 (31%) | 2.18 (0.76 to 6.21) |
| Stochino  (2020)^3^ | All Participants | All-Cause Mortality | TB and SARS-CoV-2 Co-Infected: 20  TB Only: 4 | TB and SARS-CoV-2 Co-Infected: 1 (5%)  TB Only: 1 (25%) | 0.16 (0.01 to 3.26) |
|  | Only SARS-CoV-2 diagnosed within 28 days of TB | All-Cause Mortality | TB and SARS-CoV-2 Co-Infected: 8  TB Only: 4 | TB and SARS-CoV-2 Co-Infected: 1 (13%)  TB Only: 1 (25%) | 0.43 (0.02 to 9.36) |
|  | Only SARS-CoV-2 diagnosed >28 days after TB | All-Cause Mortality | TB and SARS-CoV-2 Co-Infected: 12  TB Only: 4 | TB and SARS-CoV-2 Co-Infected: 0 (0%)  TB Only: 1 (25%) | N/A |
| Gubkina  (2020)^5^ | All Participants  (All SARS-CoV-2 diagnosed >28 days after TB) | All-Cause Mortality | TB and SARS-CoV-2 Co-Infected: 8  TB Only: 17 | TB and SARS-CoV-2 Co-Infected: 0 (0%)  TB Only: 0 (0%) | N/A |
| Gomes  (2021)^6^ | All Participants* | All-Cause Mortality | TB and SARS-CoV-2 Co-Infected: 18  TB Only: 35 | TB and SARS-CoV-2 Co-Infected: 0 (0%)  TB Only: 2 (6%) | N/A |
|  | Only SARS-CoV-2 diagnosed within 28 days of TB | All-Cause Mortality | TB and SARS-CoV-2 Co-Infected: 2  TB Only: 35 | TB and SARS-CoV-2 Co-Infected: 0 (0%)  TB Only: 2 (6%) | N/A |
|  | Only SARS-CoV-2 diagnosed >28 days before TB | All-Cause Mortality | TB and SARS-CoV-2 Co-Infected: 16  TB Only: 35 | TB and SARS-CoV-2 Co-Infected: 0 (0%)  TB Only: 2 (6%) | N/A |
| Kumar  (2021)^8^ | All Participants | All-Cause Mortality | TB and SARS-CoV-2 Co-Infected: 184  TB Only: 5225 | TB and SARS-CoV-2 Co-Infected: 35 (19%)  TB Only: 470 (9%) | 2.38 (1.62 to 3.48) |
| du Bruyn  (2021)^4^ | All Participants | In-Hospital Mortality | TB and SARS-CoV-2 Co-Infected: 15  TB Only: 5 | TB and SARS-CoV-2 Co-Infected: 6 (40%)  TB Only: 0 (0%) | N/A |
| Zulmansyah  (2021)^7^ | All Participants | In-Hospital Mortality | TB and SARS-CoV-2 Co-Infected: 16  TB Only: 13 | TB and SARS-CoV-2 Co-Infected: 1 (6%)  TB Only: 0 (0%) | N/A |

*Includes participants currently infected and seropositive in the co-infected group.

# Table F. Loss to Follow-up Among Select Populations in Each Study Evaluating TB Patients Co-infected with SARS-CoV-2 vs. TB Patients without SARS-CoV-2

| **Author**  **(Year)** | **Population Subgroup** | **Outcome** | **Participants with Known Outcome** | **Participants with Outcome (%)** | **Odds Ratio (95% CI) for Outcome if Co-Infected with SARS-CoV-2** |
| --- | --- | --- | --- | --- | --- |
| Kilic  (2022)^1^ | All Participants  (All SARS-CoV-2 diagnosed >28 days after TB) | Loss to Follow-Up | TB and SARS-CoV-2 Co-Infected: 4  TB Only: 16 | TB and SARS-CoV-2 Co-Infected: 0 (0%)  TB Only: 0 (0%) | N/A |
| Mohr-Holland  (2021)^2^ | All Participants | Loss to Follow-Up | TB and SARS-CoV-2 Co-Infected: 27  TB Only: 54 | TB and SARS-CoV-2 Co-Infected: 4 (15%)  TB Only: 11 (20%) | 0.68 (0.19 to 2.38) |
|  | Only SARS-CoV-2 diagnosed within 28 days of TB | Loss to Follow-Up | TB and SARS-CoV-2 Co-Infected: 7  TB Only: 54 | TB and SARS-CoV-2 Co-Infected: 1 (14%)  TB Only: 11 (20%) | 0.65 (0.07 to 5.99) |
|  | Only SARS-CoV-2 diagnosed >28 days after TB | Loss to Follow-Up | TB and SARS-CoV-2 Co-Infected: 20  TB Only: 54 | TB and SARS-CoV-2 Co-Infected: 3 (15%)  TB Only: 11 (20%) | 0.69 (0.17 to 2.78) |
| Stochino  (2020)^3^ | All Participants | Loss to Follow-Up | TB and SARS-CoV-2 Co-Infected: 20  TB Only: 4 | TB and SARS-CoV-2 Co-Infected: 5 (25%)  TB Only: 0 (0%) | N/A |
|  | Only SARS-CoV-2 diagnosed within 28 days of TB | Loss to Follow-Up | TB and SARS-CoV-2 Co-Infected: 8  TB Only: 4 | TB and SARS-CoV-2 Co-Infected: 2 (25%)  TB Only: 0 (0%) | N/A |
|  | Only SARS-CoV-2 diagnosed >28 days after TB | Loss to Follow-Up | TB and SARS-CoV-2 Co-Infected: 12  TB Only: 4 | TB and SARS-CoV-2 Co-Infected: 3 (25%)  TB Only: 0 (0%) | N/A |
| Gubkina  (2020)^5^ | All Participants  (All SARS-CoV-2 diagnosed >28 days after TB) | Loss to Follow-Up | TB and SARS-CoV-2 Co-Infected: 8  TB Only: 17 | TB and SARS-CoV-2 Co-Infected: 0 (0%)  TB Only: 0 (0%) | N/A |
| Gomes  (2021)^6^ | All Participants* | Loss to Follow-Up | TB and SARS-CoV-2 Co-Infected: 18  TB Only: 35 | TB and SARS-CoV-2 Co-Infected: 0 (0%)  TB Only: 2 (6%) | N/A |
|  | Only SARS-CoV-2 diagnosed within 28 days of TB | Loss to Follow-Up | TB and SARS-CoV-2 Co-Infected: 2  TB Only: 35 | TB and SARS-CoV-2 Co-Infected: 0 (0%)  TB Only: 2 (6%) | N/A |
|  | Only SARS-CoV-2 diagnosed >28 days before TB | Loss to Follow-Up | TB and SARS-CoV-2 Co-Infected: 16  TB Only: 35 | TB and SARS-CoV-2 Co-Infected: 0 (0%)  TB Only: 2 (6%) | N/A |

*Includes participants currently infected and seropositive in the co-infected group.

# Table G. Treatment Failure Among Select Populations in Each Study Evaluating TB Patients Co-infected with SARS-CoV-2 vs. TB Patients without SARS-CoV-2

| **Author**  **(Year)** | **Population Subgroup** | **Outcome** | **Participants with Known Outcome** | **Participants with Outcome (%)** | **Odds Ratio (95% CI) for Outcome if Co-Infected with SARS-CoV-2** |
| --- | --- | --- | --- | --- | --- |
| Kilic  (2022)^1^ | All Participants  (All SARS-CoV-2 diagnosed >28 days after TB) | Treatment Failure | TB and SARS-CoV-2 Co-Infected: 4  TB Only: 16 | TB and SARS-CoV-2 Co-Infected: 0 (0%)  TB Only: 0 (0%) | N/A |
| Mohr-Holland  (2021)^2^ | All Participants | Treatment Failure | TB and SARS-CoV-2 Co-Infected: 27  TB Only: 54 | TB and SARS-CoV-2 Co-Infected: 0 (0%)  TB Only: 1 (2%) | N/A |
|  | Only SARS-CoV-2 diagnosed within 28 days of TB | Treatment Failure | TB and SARS-CoV-2 Co-Infected: 7  TB Only: 54 | TB and SARS-CoV-2 Co-Infected: 0 (0%)  TB Only: 1 (2%) | N/A |
|  | Only SARS-CoV-2 diagnosed >28 days after TB | Treatment Failure | TB and SARS-CoV-2 Co-Infected: 20  TB Only: 54 | TB and SARS-CoV-2 Co-Infected: 0 (0%)  TB Only: 1 (2%) | N/A |
| Stochino  (2020)^3^ | All Participants | Treatment Failure | TB and SARS-CoV-2 Co-Infected: 20  TB Only: 4 | TB and SARS-CoV-2 Co-Infected: 0 (0%)  TB Only: 0 (0%) | N/A |
|  | Only SARS-CoV-2 diagnosed within 28 days of TB | Treatment Failure | TB and SARS-CoV-2 Co-Infected: 8  TB Only: 4 | TB and SARS-CoV-2 Co-Infected: 0 (0%)  TB Only: 0 (0%) | N/A |
|  | Only SARS-CoV-2 diagnosed >28 days after TB | Treatment Failure | TB and SARS-CoV-2 Co-Infected: 12  TB Only: 4 | TB and SARS-CoV-2 Co-Infected: 0 (0%)  TB Only: 0 (0%) | N/A |
| Gubkina  (2020)^5^ | All Participants  (All SARS-CoV-2 diagnosed >28 days after TB) | Treatment Failure | TB and SARS-CoV-2 Co-Infected: 8  TB Only: 17 | TB and SARS-CoV-2 Co-Infected: 0 (0%)  TB Only: 0 (0%) | N/A |
| Gomes  (2021)^6^ | All Participants* | Treatment Failure | TB and SARS-CoV-2 Co-Infected: 18  TB Only: 35 | TB and SARS-CoV-2 Co-Infected: 2 (11%)  TB Only: 5 (14%) | 0.75 (0.13 to 4.31) |
|  | Only SARS-CoV-2 diagnosed within 28 days of TB | Treatment Failure | TB and SARS-CoV-2 Co-Infected: 2  TB Only: 35 | TB and SARS-CoV-2 Co-Infected: 0 (0%)  TB Only: 5 (14%) | N/A |
|  | Only SARS-CoV-2 diagnosed >28 days before TB | Treatment Failure | TB and SARS-CoV-2 Co-Infected: 16  TB Only: 35 | TB and SARS-CoV-2 Co-Infected: 2 (13%)  TB Only: 5 (14%) | 0.86 (0.15 to 4.97) |

*Includes participants currently infected and seropositive in the co-infected group.

# Table H. Evidence Profile

1. **Coinfection Review**

| **Certainty assessment** | | | | | | | **№ of patients** | | **Certainty** | **Importance** |
| --- | --- | --- | --- | --- | --- | --- | --- | --- | --- | --- |
| **№ of studies** | **Study design** | **Risk of bias** | **Inconsistency** | **Indirectness** | **Imprecision** | **Other considerations** | **TB Disease Alone** | **TB Disease with SARS-CoV-2 Infection** |  |  |
| **All Unfavorable TB Outcomes** | | | | | | | | | | |
| 5 ^a^ | observational studies | very serious ^b^ | serious ^c^ | very serious ^d^ | very serious ^e^ | none | TB Alone (6 studies, 110 patients): Proportion with outcome ranges from 0% to 63%  TB with SARS-CoV-2 (6 studies, 93 patients): Proportion with outcome ranges from 0% to 67%.  Odds ratios (estimable in 3 studies) range from 0.36 to 1.29; in 2 studies where not estimable, unfavorable outcomes were equivalent (0% in both groups) in both studies. | | ⨁◯◯◯ VERY LOW | CRITICAL |
| **All-Cause or In-Hospital Mortality** | | | | | | | | | | |
| 8 ^a^ | observational studies | very serious ^f^ | very serious ^g^ | very serious ^d^ | very serious ^e^ | none | TB Alone (8 studies, 5353 patients): Proportion with outcome ranges from 0% to 31%  TB with SARS-CoV-2 (8 studies, 308 patients): Proportion with outcome ranges from 0% to 48%.  Odds ratios (estimable in 3 studies) range from 0.16 to 2.38; in 5 studies where not estimable, more mortality seen in co-infected group in two studies, in TB only group in one study, and equivalent (0% in both groups) in two studies. | | ⨁◯◯◯ VERY LOW | CRITICAL |
| **Loss to Follow-Up** | | | | | | | | | | |
| 5 ^a^ | observational studies | very serious ^b^ | serious ^c^ | very serious ^d^ | very serious ^e^ | none | TB Alone (5 studies, 110 patients): Proportion with outcome ranges from 0% to 20%  TB with SARS-CoV-2 (5 studies, 93 patients): Proportion with outcome ranges from 0% to 25%.  Odds ratio (estimable in 1 study) = 0.68 (95% CI: 0.19 to 2.38); in 4 studies where not estimable, more lost to follow-up seen in co-infected group in one study, in TB only group in one study, and equivalent (0% in both groups) in two studies. | | ⨁◯◯◯ VERY LOW | CRITICAL |
| **Treatment Failure** | | | | | | | | | | |
| 6 ^a^ | observational studies | very serious ^b^ | serious ^c^ | very serious ^d^ | very serious ^e^ | none | TB Alone (6 studies, 110 patients): Proportion with outcome ranges from 0% to 14%  TB with SARS-CoV-2 (6 studies, 93 patients): Proportion with outcome ranges from 0% to 11%.  Odds ratio (estimable in 1 study) = 0.75 (95% CI 0.13 to 4.31); in 4 studies where not estimable, more treatment failure seen in TB only group in one study, and equivalent (0% in both groups) in three studies. | | ⨁◯◯◯ VERY LOW | CRITICAL |

1. Denominators include only those with known SARS-CoV-2 infection status.
2. Risk of bias considered very serious as no study designed or aimed to answer the review question, representativeness of exposure groups was generally poor due to setting and selection criteria for testing, significant differences in exposure and comparator populations existed, absence of adjustment for estimates of effect, and unclear differences in management and care.
3. Inconsistency considered serious as direction of estimated effect varied among studies where this was estimable.
4. Very serious indirectness due to applicability concerns (most studies done early in the SARS-CoV-2 pandemic), selected nature of participants (mostly inpatients), and lack of any identified study having the primary aim to answer the review question.
5. Very serious imprecision caused by small sample sizes and very wide confidence intervals where estimates of effect could be calculated.
6. Risk of bias considered very serious as the representativeness of exposure groups was generally poor due to setting and selection criteria for testing, significant differences in exposure and comparator populations existed, absence of adjustment for estimates of effect, unclear differences in management and care, differential follow-up times for outcome ascertainment (up to hospital discharge or up to end of treatment), and potential for misclassification bias.
7. Inconsistency considered very serious as directions of effect ranged from SARS-CoV-2 being protective to being harmful, with some studies only reporting deaths in only one population group; one study considered SARS-CoV-2 to be significantly harmful.
8. **Clinical Management Review**

| **Certainty assessment** | | | | | | | **№ of patients** | | **Certainty** | **Importance** |
| --- | --- | --- | --- | --- | --- | --- | --- | --- | --- | --- |
| **№ of studies** | **Study design** | **Risk of bias** | **Inconsistency** | **Indirectness** | **Imprecision** | **Other considerations** | **No Steroid or Immunomodulating Treatment** | **Received Steroid or Immunomodulating Treatment** |  |  |
| **All Unfavorable TB Outcomes** | | | | | | | | | | |
| 1 ^a^ | observational studies | very serious ^b^ | serious ^c^ | very serious ^d^ | very serious ^e^ | none | No Treatment (1 study, 95 participants): 36 (38%) with outcome  Treatment (1 study, 23 participants): 20 (87%) with outcome  Crude odds ratio: 10.93 (3.03 to 39.39) | | ⨁◯◯◯ VERY LOW | CRITICAL |
| **Severe/Critical COVID-19 or Mortality**^f^ | | | | | | | | | | |
| 1 ^a^ | observational studies | very serious ^g^ | very serious ^h^ | very serious ^i^ | very serious ^e^ | none | No Treatment (1 study, 689 participants): 541 (79%) with outcome  Treatment (1 study, 117 participants): 60 (51%) with outcome  Adjusted odds ratio: 1.00 (0.59 to 1.70) | | ⨁◯◯◯ VERY LOW | CRITICAL |
| **All-Cause or In-Hospital Mortality** | | | | | | | | | | |
| 4 ^a^ | observational studies | very serious ^j^ | very serious ^k^ | very serious ^l^ | very serious ^e^ | none | No Treatment (4 studies, 1281 participants): Proportion with outcome ranges from 14% to 73%.  Treatment (4 studies, 291 participants): Proportion with outcome ranges from 13% to 83%.  Crude odds ratios in two studies of 7.09 (95% CI 2.41 to 20.83) and 10.00 (95% CI 0.78 to 128.77); adjusted odds ratio in one study of 1.11 (95% CI 0.66 to 1.87); adjusted hazard ratio in one study of 0.49 (0.30 to 0.79). | | ⨁◯◯◯ VERY LOW | CRITICAL |
| **Mechanical Ventilation** | | | | | | | | | | |
| 2 ^a^ | observational studies | very serious ^m^ | very serious ^n^ | very serious ^l^ | very serious ^e^ | none | No Treatment (2 studies, 771 participants): Proportion with outcome ranges from 2% to 20%.  Treatment (2 studies, 140 participants): Proportion with outcome ranges from 2% to 48%.  Crude odds ratios in one study of 3.78 (95% CI 1.41 to 10.11); adjusted odds ratio in one study of 0.61 (95% CI 0.08 to 2.68). | | ⨁◯◯◯ VERY LOW | CRITICAL |
| **Intensive Care Unit Admission** | | | | | | | | | | |
| 1 ^a^ | observational studies | very serious ^g^ | very serious ^o^ | very serious ^i^ | very serious ^e^ | none | No Treatment (1 study, 689 participants): 49 (7%) with outcome  Treatment (1 study, 117 participants): 4 (3%) with outcome  Adjusted odds ratio: 0.70 (0.20 to 2.00) | | ⨁◯◯◯ VERY LOW | CRITICAL |

1. Denominators include only those with known SARS-CoV-2 infection status.
2. Risk of bias considered very serious as the included study consisted of patients from 54 centres in 24 countries from early in the SARS-CoV-2 pandemic, steroid use is likely strongly confounded by indication as the proportion of participants receiving steroids using supplemental oxygen was 86% compared to 29% among those without steroids, timing of steroid initiation is unknown, and there are likely significant and unknown differences in management and care between exposure and comparator groups.
3. Inconsistency considered serious as strength in crude associations varied by time between SARS-CoV-2 diagnosis and TB treatment initiation.
4. Very serious indirectness due to applicability concerns (all participants enrolled early in the SARS-CoV-2 pandemic), selected nature of participants (mostly inpatients and from centres with an average number of participants of approximately 2), and that all contributing studies did not have the primary aim to answer the review question.
5. Very serious imprecision caused by small sample sizes and very wide confidence intervals where estimates of effect could be calculated.
6. Defined as intensive care unit admission, mechanical ventilation, and/or death.
7. Risk of bias considered very serious as included study did not have timing of steroid initiation or of any primary outcomes (i.e., ventilation, ICU admission), which may lead to the outcome preceding steroid use, risk of immortal time bias, where those who survive are more likely to receive steroids, risk of confounding by indication, the significant differences in exposure and comparator populations, unclear differences in management and care, and potential for misclassification bias.
8. Very serious inconsistency as direction of effect varied in subgroup analyses and by component outcomes (from potentially harmful to potentially beneficial).
9. Very serious indirectness due to applicability concerns (single country study with high HIV prevalence and unknown prevalence of drug-resistant tuberculosis in the included population) and that the initial study was not designed to answer this review question.
10. Very serious risk of bias as included studies had varying follow-up times (to end of TB treatment or hospital discharge), risk of immortal time bias (those who survive are more likely to receive steroids), significant differences in exposure and comparator populations, risk of confounding by indication, unclear differences in management and care, and potential for misclassification bias.
11. Inconsistency considered very serious as direction of effect varied across included studies and by analytical method, as well as varying in subgroup analyses.
12. Indirectness considered very serious due to secondary use of data, applicability concerns due to care practices, circulating variants, and background immunity, and selected nature of participants in some studies.
13. Risk of bias considered very serious due to steroid use being likely strongly confounded by indication, unknown timing of steroid initiation in the disease course, and likely significant and unknown differences in management and care between exposure and comparator groups.
14. Very serious inconsistency with directions of effect being different between studies and among subgroups analyzed.
15. Very serious inconsistency due to direction of effect varying during subgroup analyses from potentially strongly protective to being harmful.
