# Supplementary material for: The association of SARS-CoV-2 infection and tuberculosis disease with unfavorable treatment outcomes: A systematic review
Source: PLOS Glob Public Health. 2023 Jul 19;3(7):e0002163. doi: 10.1371/journal.pgph.0002163 (PMC10355446; doi:10.1371/journal.pgph.0002163)
Supplement: S2 Text — (DOCX) [file pgph.0002163.s002.docx]

# S2 Text. Additional Details for Each Study as Supplied by Authors

*Kilic (2022)^1^*

- All 20 participants with a valid RT-PCR test reported in the original study are included.
- The authors provided treatment outcome information for all 20 participants (all treatment success).
- The authors confirmed no person testing positive for SARS-CoV-2 via RT-PCR received steroids or immunomodulating treatment.
- The authors provided the time from TB treatment initiation for the 4 RT-PCR positive participants (range: 99-161 days after initiation).
- The authors provided the age and sex distribution of the 4 RT-PCR positive participants (male age 66, male age 41, male age 23, and female age 49)
- The authors provided the age and sex distribution of the 16 RT-PCR negative participants (median age 53, 14 males, 2 females)
- The authors stated the reason for offering COVID-19 testing was: “Having symptoms and radiological findings consistent with COVID-19 pneumonia and/or a history of close contact with patients diagnosed with COVID-19.”
- They confirmed that the person hospitalized in the study was negative for SARS-CoV-2 by RT-PCR but had infiltrations on CT scan that were consistent with COVID-19 pneumonia.

*Mohr-Holland (2021)^2^*

- The original publication included data from April to November 2020 with 75 patients being tested for COVID-19. The shared cohort is an update of the originally reported cohort, now including participants with at least 2 months of treatment who received a COVID-19 test from March 2020 through June 2021, inclusive. The cohort comprises 103 SARS-CoV-2 negative rifampicin-resistant TB patients, 8 SARS-CoV-2 positive rifampicin-resistant TB patients diagnosed within 28 days of tuberculosis, and 28 SARS-CoV-2 positive rifampicin-resistant TB patients diagnosed >28 days after tuberculosis.
- The additional data – as supplied by the study team – are provided in the subsequent three tables.

| **All data combined** | | | | |
| --- | --- | --- | --- | --- |
| Characteristic | No SARS-CoV-2 (Group 1) | SARS-CoV-2 | | Total |
|  |  | Diagnosed between 28d before and 28d after TB diagnosis  (Group 2) | Diagnosed >28d after TB diagnosis  (Group 3) |  |
| N people | 103 | 8 | 28 | 139 |
| Median Age (IQR) | 35 (30-46) | 43 (33-65) | 36 (31-56) | 35 (30-48) |
| Female Sex | 52 (50%) | 4 (50%) | 11 (39%) | 67 (48%) |
| HIV Characteristics |  |  |  |  |
| Living with HIV | 79 (77%) | 7 (88%) | 17 (61%) | 103 (74%) |
| TB Tx History |  |  |  |  |
| No previous TB tx | 46 (45%) | 2 (25%) | 14 (50%) | 62 (45%) |
| Previous TB tx with FLD only | 47 (46%) | 5 (63%) | 12 (43%) | 64 (46%) |
| Previous TB tx with SLD | 10 (10%) | 1 (13%) | 2 (7%) | 13 (9%) |
| Site of disease |  |  |  |  |
| Pulmonary TB | 94 (91%) | 5 (63%) | 24 (86%) | 123 (88%) |
| EPTB only | 9 (9%) | 3 (38%) | 4 (14%) | 16 (12%) |
| Resistance Patterns |  |  |  |  |
| Xpert RR-TB only | 10 (10%) | 0 (0%) | 7 (25%) | 17 (12%) |
| RMR-TB | 24 (23%) | 2 (25%) | 6 (21%) | 32 (23%) |
| MDR-TB | 48 (47%) | 5 (63%) | 7 (25%) | 60 (43%) |
| MDR+ INJ-R | 4 (4%) | 0 (0%) | 0 (0%) | 4 (3%) |
| MDR +FQ-R | 9 (9%) | 1 (13%) | 5 (18%) | 15 (11%) |
| Unconfirmed RR-TB |  |  |  |  |
| MDR+INJ+FLQ | 2 (2%) | 0 (0%) | 1 (4%) | 3 (2%) |
| Unconfirmed RR-TB | 6 (6%) | 0 (0%) | 2 (7%) | 8 (6%) |
| TB Outcomes |  |  |  |  |
| TB Tx Success | 20 (37%) | 3 (43%) | 6 (30%) | 29 (36%) |
| TB Tx Failure | 1 (2%) | 0 (0%) | 0 (0%) | 1 (1%) |
| TB Tx LTFU | 11 (20%) | 1 (14%) | 3 (15%) | 15 (19%) |
| Died | 17 (31%) | 3 (43%) | 10 (50%) | 30 (37%) |
| Still on Tx | 49 (48%) | 1 (13%) | 8 (29%) | 58 (42%) |
| Other (transfer) | 5 (9%) | 0 (0%) | 1 (5%) | 6 (7%) |
| Sputum culture conversion by month 2 (among still on Tx) | 27/38  Remainder missing cultures | 1/1 | 5/7  Remainder missing cultures | 33/46 |

| **HIV negative** | | | | |
| --- | --- | --- | --- | --- |
| Characteristic | No SARS-CoV-2  (Group 1) | SARS-CoV-2 | | Total |
|  |  | Diagnosed between 28d before and 28d after TB diagnosis  (Group 2) | Diagnosed >28d after TB diagnosis  (Group 3) |  |
| N people | 23 | 1 | 11 | 35 |
| Median Age (IQR) |  |  |  |  |
| Female Sex | 9 (39%) | 1 (100%) | 1 (9%) | 11 (31%) |
| TB Tx History |  |  |  |  |
| No previous TB tx | 14 (61%) | 0 (0%) | 10 (91%) | 24 (69%) |
| Previous TB tx with FLD only | 5 (22%) | 1 (100%) | 1 (9%) | 7 (20%) |
| Previous TB tx with SLD | 4 (17%) | 0 (0%) | 0 (0%) | 4 (11%) |
| Site of disease |  |  |  |  |
| Pulmonary TB | 22 (96%) | 1 (100%) | 11 (100%) | 34 (97%) |
| EPTB only | 1 (4%) | 0 (0%) | 0 (0%) | 1 (3%) |
| Resistance Patterns |  |  |  |  |
| Xpert RR-TB only | 4 (17%) | 0 (0%) | 3 (27%) | 7 (20%) |
| RMR-TB | 2 (9%) | 0 (0%) | 2 (18%) | 4 (11%) |
| MDR-TB | 11 (48%) | 0 (0%) | 2 (18%) | 13 (37%) |
| MDR+ INJ-R | 1 (4%) | 0 (0%) | 0 (0%) | 1 (3%) |
| MDR +FQ-R | 3 (13%) | 1 (100%) | 4 (36%) | 8 (23%) |
| MDR+INJ+FLQ | 0 (0%) | 0 (0%) | 0 (0%) | 0 (0%) |
| Unconfirmed RR-TB | 2 (9%) | 0 (0%) | 0 (0%) | 2 (6%) |
| TB Outcomes |  |  |  |  |
| TB Tx Success | 5 (38%) | 0 (0%) | 4 (50%) | 9 (41%) |
| TB Tx Failure | 0 (0%) | 0 (0%) | 0 (0%) | 0 (0%) |
| TB Tx LTFU | 3 (23%) | 0 (0%) | 0 (0%) | 3 (14%) |
| Died | 3 (23%) | 1 (100%) | 4 (50%) | 8 (36%) |
| Still on Tx | 10 (43%) | 0 (0%) | 3 (27%) | 13 (37%) |
| Other (transfer) | 2 (15%) | 0 (0%) | 0 (0%) | 2 (9%) |
| Sputum culture conversion by month 2 (among still on Tx) | 5/7 |  | 1/3 | 6/10 |

| **PLHIV** | | | | |
| --- | --- | --- | --- | --- |
| Characteristic | No SARS-CoV-2  (Group 1) | SARS-CoV-2 | | Total |
|  |  | Diagnosed between 28d before and 28d after TB diagnosis  (Group 2) | Diagnosed >28d after TB diagnosis  (Group 3) |  |
| N people | 79 | 7 | 17 | 103 |
| Median Age (IQR) |  |  |  |  |
| Female Sex | 42 (53%) | 3 (43%) | 6 (35%) | 51 (50%) |
| If HIV, on ART | 72 (91%) | 5 (71%) | 15 (88%) | 92 (89%) |
| Baseline CD4 <200 | 48 (61%) | 5 (71%) | 12 (71%) | 65 (63%) |
| Baseline CD4 200-499 | 9 (11%) | 1 (14%) | 0 (0%) | 10 (10%) |
| Baseline CD4 >=500 | 4 (5%) | 0 (0%) | 0 (0%) | 4 (4%) |
| Baseline CD4 Unknown | 18 (23%) | 1 (14%) | 5 (29%) | 24 (23%) |
| TB Tx History |  |  |  |  |
| No previous TB tx | 31 (39%) | 2 (29%) | 4 (24%) | 37 (36%) |
| Previous TB tx with FLD only | 42 (53%) | 4 (57%) | 11 (65%) | 57 (55%) |
| Previous TB tx with SLD | 6 (8%) | 1 (14%) | 2 (12%) | 9 (9%) |
| Site of disease |  |  |  |  |
| Pulmonary TB | 71 (90%) | 4 (57%) | 13 (76%) | 88 (85%) |
| EPTB only | 8 (10%) | 3 (43%) | 4 (24%) | 15 (15%) |
| Resistance Patterns |  |  |  |  |
| Xpert RR-TB only | 6 (8%) | 0 (0%) | 4 (24%) | 10 (10%) |
| RMR-TB | 22 (28%) | 2 (29%) | 4 (24%) | 28 (27%) |
| MDR-TB | 36 (46%) | 5 (71%) | 5 (29%) | 46 (45%) |
| MDR+ INJ-R | 3 (4%) | 0 (0%) | 0 (0%) | 3 (3%) |
| MDR +FQ-R | 6 (8%) | 0 (0%) | 1 (6%) | 7 (7%) |
| MDR+INJ+FLQ | 2 (3%) | 0 (0%) | 1 (6%) | 3 (3%) |
| Unconfirmed RR-TB | 4 (5%) | 0 (0%) | 2 (12%) | 6 (6%) |
| TB Outcomes |  |  |  |  |
| TB Tx Success | 15 (38%) | 3 (50%) | 2 (17%) | 20 (34%) |
| TB Tx Failure | 1 (3%) | 0 (0%) | 0 (0%) | 1 (2%) |
| TB Tx LTFU | 8 (18%) | 1 (17%) | 3 (25%) | 11 (19%) |
| Died | 14 (35%) | 2 (33%) | 6 (50%) | 22 (38%) |
| Still on Tx | 39 (49%) | 1 (14%) | 5 (29%) | 45 (44%) |
| Other (transfer) | 3 (8%) | 0 (0%) | 1 (8%) | 4 (7%) |
| Sputum culture conversion by month 2 (among still on Tx) | 22/31 | 1/1 | 4/4 | 27/36 |

*Stochino (2020)^3^*

- The authors provided the study team with additional information on the 24 TB patients in their study, of whom 20 were coinfected with SARS-CoV-2.
- For the 4 patients with only TB disease, the authors provided the median age (27 years), sex distribution (2 females, 2 males), body mass index information (1 underweight, 3 normal), TB location (all had pulmonary TB and 2 had extrapulmonary involvement), drug-resistance (1 had multidrug-resistant TB), and TB outcomes (3 successfully treated, and one died of AIDS).
- For the 20 patients with TB/SARS-CoV-2, the authors provided treatment outcome data in the table below.

| **Pts.** | **Age** | **Sex** | **Progress to severe/critical COVID-19** | **Received steroids/ immunomodulating treatment** | **Time Since TB (days)** | **Died of COVID-19** | **End of TB treatment outcome** (success, failure, death, loss to follow-up, still on treatment) |
| --- | --- | --- | --- | --- | --- | --- | --- |
| P01 | 20s | M | No | No | 20 | No | Success |
| P02 | 60s | M | No | No | 84 | No | Success |
| P03 | 10s | F | No | No | 20 | No | Lost to follow-up |
| P04 | 20s | M | No | No | 14 | No | Lost to follow-up |
| P05 | 30s | F | No | No | 302 | No | Lost to follow-up |
| P06 | 70s | F | Yes | No | 26 | Yes | Death |
| P07 | 30s | M | No | No | 21 | No | Success |
| P08 | 20s | M | No | No | 19 | No | Success |
| P09 | 40s | M | No | No | 6 | No | Success |
| P10 | 40s | F | No | No | 8 | No | Success |
| P11 | 20s | M | No | No | 53 | No | Success |
| P12 | 60s | F | No | No | 56 | No | Lost to follow-up |
| P13 | 40s | F | No | No | 152 | No | Success |
| P14 | 60s | M | No | No | 62 | No | Success |
| P15 | 30s | M | No | No | 97 | No | Lost to follow-up |
| P16 | 40s | M | No | No | 43 | No | Success |
| P17 | 20s | F | No | No | 38 | No | Success |
| P18 | 30s | M | No | No | 30 | No | Success |
| P19 | 20s | F | No | No | 87 | No | Success |
| P20 | 30s | M | No | No | 40 | No | Success |

*Du Bruyn (2021)^4^*

- The authors provided detailed information on the 15 participants with TB/COVID-19 detailed in Supplement Table 3 of their original publication, plus information on 5 hospitalized control patients with TB disease only.
- The authors provided more specific detail on the age of the participants, as well as whether participants received steroids (none received other immunomodulating treatments). Specific detail supplied by the authors are in the table below.

| **Patient number** | **Age** | **HIV-1 status** | **CD4 /mm^3^** | **Viral Load** | **on ART Y/N** | **Method of TB diagnosis** | **Time between + SARS CoV-2 PCR and TB dx** | **Prior TB episodes** | **Time since last TB episode** | **WHO score at enrolment** | **Outcome** | **On steroids** |
| --- | --- | --- | --- | --- | --- | --- | --- | --- | --- | --- | --- | --- |
| **COVID-19/TB** | | | | | |  |  |  |  |  |  |  |
| 9 | 37 | -ve | NA | NA | NA | sputum Xpert + (Rif S) | simultaneous | 2 | 3 years | 4 | discharged | N |
| 36 | 43 | -ve | NA | NA | NA | sputum Xpert + (Rif S) | TB dx 25 days after | 0 | NA | 5 | discharged | Y |
| 57 | 37 | -ve | NA | NA | NA | sputum culture + (after 24 days; sputum Xpert ND) | TB dx 6 weeks prior | 0 | NA | 3 | discharged | N |
| 58 | 43 | -ve | NA | NA | NA | sputum Xpert + (Rif S) | simultaneous | 0 | NA | 4 | discharged | Y |
| 73 | 41 | -ve | NA | NA | NA | sputum Xpert + (Rif R) | simultaneous | 0 | NA | 6 | died | Y |
| 80 | 61 | -ve | NA | NA | NA | sputum Xpert + (Rif S) | TB dx 20 days after | 0 | NA | 7 | died | Y |
| 125 | 65 | -ve | NA | NA | NA | sputum Xpert + (Rif S) | simultaneous | 0 | NA | 6 | died | Y |
| 3 | 31 | +ve | 106 | ND | N | pleural fluid Xpert + (Rif S) | simultaneous | 0 | NA | 3 | discharged | N |
| 6 | 34 | +ve | 110 | 17870 | Y | sputum Xpert + (Rif S) | TB dx 3 months prior | 0 | NA | 4 | discharged | N |
| 61 | 37 | +ve | 26 | 523463 | Y | clinical diagnosis: disseminated TB | TB dx 18 days prior | 1 | 2 years | 4 | died | Y |
| 93 | 43 | +ve | 51 | 2941 | Y | sputum Xpert + (Rif S), sputum auramine 3+ | simultaneous | 1 | 4 months | 4 | died | N |
| 95 | 49 | +ve | 106 | 15860 | N | sputum Xpert + (Rif S) and culture positive (Rif S) | simultaneous | 0 | NA | 3 | discharged | N |
| 97 | 55 | +ve | 17 | 201574 | N | pericardial fluid and sputum Xpert sputum Xpert + (Rif S); urine LAM + | simultaneous | 0 | NA | 3 | discharged | N |
| 104 | 56 | +ve | 209 | LDL | Y | sputum Xpert + (Rif S) | TB dx 21 days after | 0 | NA | 5 | died | Y |
| 152 | 27 | +ve | ND | 395 | Y | urine LAM positive, urine Mtb culture positive (TTP 20 days, Rif R, INH S) | simultaneous | 2 | 1 year | 3 | discharged | Y |
| **Hospitalized controls (TB Only)** | | | | | |  | **Time between admission and TB diagnosis** |  |  |  |  |  |
| 52 | 31 | +ve | ND | 2352 | Y | sputum Xpert positive | TB dx 6 months prior | 0 | NA | 3 | discharged | N |
| 77 | 29 | +ve | ND | 40 | Y | clinical | TB dx 3 months prior | 0 | NA | 3 | discharged | N |
| 136 | 45 | +ve | 18 | 273385 | N | sputum Xpert positive | diagnosed on admission | 0 | NA | 3 | discharged | N |
| 144 | 42 | +ve | 5 | 232030 | Y | clinical | TB dx 5 months prior | 1 | 3 years | 4 | discharged | Unknown |
| 151 | 55 | +ve | 17 | 344470 | N | sputum Xpert positive | diagnosed on admission | 1 | 4 years | 4 | discharged | Y |

- The mean WHO score at enrollment among coinfected participants = 4.27
  - Among those on steroids = 5.0
  - Among those without steroids = 3.42
- The mean WHO score at enrollment among TB only participants = 3.40

*Gubkina (2020)^5^*

- The cohort of participants included in the additional data supplied by the authors is identical to the cohort reported in the original publication.
- The authors confirmed that no children in the study received steroids or immunomodulating treatments.
- The authors relayed information on that all children in the study completed treatment and were discharged in a satisfactory condition being clinically cured with stable chest x-rays.
- For those with TB/COVID-19, all children received a COVID-19 diagnosis >28 days after their initial diagnosis.
- As antibody testing and RT-PCR testing occurred >2 months into TB treatment, we assumed that any child positive via PCR or antibody was infected >28 days after TB treatment initiation.

*Gomes (2021)^6^*

- The data contributed by the authors consists of 53 of the 83 participants reported in the original study, for whom TB treatment outcome data was available (overall, 64% of participants included).
- The authors shared details on participants by SARS-CoV-2 status: negative on antibody and PCR, PCR positive, only antibody positive. The details are in the below table.

| **Characteristic** | **TB, no COVID-19** | **TB, only seropositive** | **TB, RT-PCR Positive** | **Total** |
| --- | --- | --- | --- | --- |
| N participants | 59 | 21 | 3 | 83 |
| Median (IQR) Age, years | 54.5(35) | 42 (40.5) | 33(18) | 54.5 (35) |
| Female Sex, N (%) | 25 (42%) | 6 (29%) | 1 (33%) | 40% |
| HIV Positive, N (%) | 4 (6.8%) | 2 (9.5%) | 0 (0%) | 6 (14%) |
| Pregnant, N (%) | 1 (1.7%) | 0 (0%) | 0 (0%) | 1 (2.3%) |
| Heart Disease, N (%) | 1 (1.7%) | 0 (0%) | 0 (0%) | 1 (2.3%) |
| Hypertension, N (%) | 2 (3.4%) | 1 (4.8%) | 0 (0%) | 3 (6.8%) |
| Diabetes, N (%) | 9 (15%) | 2 (9.5%) | 1 (33%) | 12 (27%) |
| Kidney Disease, N (%) | 3 (5.1%) | 0 (0%) | 0 (0%) | 3 (6.8%) |
| Neurological Disease, N (%) | 0 (0%) | 1 (4.8%) | 0 (0%) | 1 (1.2%) |
| Cancer, N (%) | 1 (1.7%) | 0 (0%) | 0 (0%) | 1 (2.3%) |
| Smoking, N (%) | 5 (8.5%) | 1 (4.8%) | 0 (0%) | 6 (14%) |
| **TB Features** |  |  |  |  |
| Pulmonary TB, N (%) | 49 (83%) | 21 (100%) | 3 (100%) | 73 (88%) |
| DS-TB, N (%) | 17 (29%) | 4 (19%) | 1 (33%) | 22 (27%) |
| RR/MDR/XDR-TB, N (%) | 35 (59%) | 11 (52%) | 2 (67%) | 48 (58%) |
| Other DR-TB, N (%) | 7 (12%) | 6 (29%) | 0 (0%) | 13 (16%) |
| New TB Case, N (%) | 40 (68%) | 15 (71%) | 3 (100%) | 58 (70%) |
| **COVID-19 Features** |  |  |  |  |
| Self-Report Previous COVID-19, N (%) | 2 (3.4%) | 1 (4.8%) | 1 (33%) | 4 (4.8%) |
| Self-Report Previous Serological Test, N (%) | 16 (27%)*  2 (3.4%)** | 5 (24%)*  1 (4.8%)** | 0 (0%) | 21 (25%)*  3 (3.6%)** |
| Time from TB Diagnosis to positive RT-PCR | N/A | N/A | 1 (33%) – 7 months  2 (67%) – 3 months | N/A |
| **TB Outcomes** |  |  |  |  |
| Outcome Known, N (%) | 35/59 (59%) | 16/21 (76%) | 2/3 (67%) | 53/83 (64%) |
| Success, N (%) | 26/35 (74%) | 14/16 (88%) | 2/2 (100%) | 42/53 (79%) |
| Failure, N (%) | 5/35 (14%) | 2/16 (12%) | 0/2 (0%) | 7/53 (13%) |
| Loss to Follow-up, N (%) | 2/35 (6%) | 0/16 (0%) | 0/2 (0%) | 2/53 (4%) |
| Died, N (%) | 2/35 (6%) | 0/16 (0%) | 0/2 (0%) | 2/53 (4%) |

* Self-Report Previous Serological Test regardless of the result

** Self-Report Previous Serological Test of who self-reported having been COVID-19 diagnosed.

*Zulmansyah (2021)^7^*

- The participants included in this study are the 29 participants with tuberculosis reported in Table 2 of the original publication.
- The author confirmed that the age range for those with COVID-19 coinfection was 26-64 years old and for those without COVID-19, it was 18-86 years. The author did not respond to a follow-up email for more specificity in age (i.e., median, IQR). The author did not respond to a query regarding use of steroids or immunomodulating treatments among those with COVID-19 coinfection.
- The author confirmed the average length of stay among those coinfected was 17.25 days and among those without COVID-19 it was 12.89 days.
- The author said only one patient died before discharge from the hospital—this patient had TB/COVID-19 coinfection. The author also added details that 3 other TB/COVID-19 patients were admitted to the ICU, however they survived.

*Kumar (2021)^8^*

- The original publication included 177 patients with TB and COVID-19 coinfection. Upon contact with the author, they agreed to share updated data from the TB database from October 2020 to March 2021 inclusive.
- Details on use of steroids or immunomodulating treatments were unavailable, including dates of diagnosis of COVID-19 and TB, which required linkage with a COVID-19 registry.
- The authors provided information that the median age of participants included was 50 years and that 65.5% were male.
- The authors shared data on 5409 participants with TB and known COVID-19 result. Only the outcome of death was recorded (other unfavorable treatment outcomes are unknown). The original table shared by the authors is below.

| **COVID-Status** | **Total Patients with TB** | **Death as Outcome** | **Proportion Dying** |
| --- | --- | --- | --- |
| Negative | 5225 | 470 | 9% |
| Positive | 184 | 35 | 17% |
| Unknown / Not Tested | 5668 | 436 | 7.7% |

*Jassat (2021)^9^*

- The data provided to the study team are an extension of the published study spanning all patients with either current or past tuberculosis admitted to participating hospitals between March 5, 2020, and March 28, 2022.
- There is no data on SARS-CoV-2 vaccination.
- The entire shared dataset consists of 13,232 patients. We excluded any individual admitted prior to October 1, 2020, as this was approximately 3 months after steroids had been recommended and widely available and therefore would reasonably be available to all patients. We further only retained adults with current tuberculosis who were at least 18 years of age, who had known discharge status, were placed into the general ward upon hospital admission, were not receiving steroids or immunosuppressants upon admission, and did not require supplemental oxygen or mechanical ventilation upon admission.
- Clinical guidelines state that steroid treatment (e.g., with dexamethasone) be reserved for patients requiring supplemental oxygen or mechanical ventilation (i.e., at a minimum severe COVID-19 disease)—see: <https://www.nicd.ac.za/diseases-a-z-index/disease-index-covid-19/covid-19-guidelines/clinical-management-of-suspected-or-confirmed-covid-19-disease/>. Therefore, we further restricted the population to only hospitalized patients with severe disease. This left 806 patients for analysis (117 receiving steroid or immunomodulating treatment and 689 without). The characteristics and outcomes of patients is included in the table below.

| Characteristic | No Tx | Tx | Total |
| --- | --- | --- | --- |
| N | 689 | 117 | 806 |
| Median (IQR) Age, y | 44 (34 to 56) | 41 (35 to 54) | 44 (34 to 56) |
| Age 18-39y | 268 (39%) | 46 (39%) | 314 (39%) |
| Age 40-59y | 278 (40%) | 54 (46%) | 332 (41%) |
| Age 60+ y | 143 (21%) | 17 (15%) | 160 (20%) |
| Female Sex | 311 (45%) | 55 (47%) | 366 (45%) |
| PLHIV | 358 (52%) | 77 (66%) | 435 (54%) |
| If PLHIV, known on ART | 128 (19%) | 58 (50%) | 186 (23%) |
| Previous TB Disease | 382 (55%) | 17 (15%) | 399 (50%) |
| Received Supplemental O_2_ | 179 (26%) | 82 (70%) | 261 (32%) |
| Discharge Status |  |  |  |
| Alive | 187 (27%) | 61 (52%) | 248 (31%) |
| Died | 502 (73%) | 56 (48%) | 558 (69%) |
| ICU Admission |  |  |  |
| Yes | 49 (7%) | 4 (3%) | 53 (7%) |
| No | 640 (93%) | 113 (97%) | 753 (93%) |
| Ventilated |  |  |  |
| Yes | 13 (2%) | 2 (2%) | 15 (2%) |
| No | 676 (98%) | 115 (98%) | 791 (98%) |
| All Negative Outcomes |  |  |  |
| Yes | 541 (79%) | 60 (51%) | 601 (75%) |
| No | 148 (21%) | 57 (49%) | 205 (25%) |

- The authors noted potential limitations in access to ICU beds and/or mechanical ventilation in the public sector in South Africa and that the overall proportion of persons experiencing these outcomes may not represent the proportion of persons requiring an ICU bed and/or mechanical ventilation.
- Analyses were adjusted for age, sex, HIV/ART, previous TB history, and receipt of supplemental oxygen (the latter being strongly associated with steroid or immunomodulating treatment use). Adjusted estimates for each outcome in the total population and by HIV status are reported in Table 3 of the main text.
- Adjusted estimates for all other subgroups analyzed are included in the table below.

| **Outcome** | **Subgroup** | **aOR (95% CI)** |
| --- | --- | --- |
| All Negative Outcomes | Age <60y | 1.03 (0.58 to 1.85) |
|  | Age ≥60y | 0.67 (0.17 to 2.58) |
|  | Male | 0.57 (0.28 to 1.17) |
|  | Female | 1.64 (0.71 to 3.86) |
|  | Previous TB History | 0.35 (0.05 to 2.14) |
|  | No Previous TB History | 0.95 (0.46 to 1.96) |
|  | Beta Wave (October 1, 2020 to April 30, 2021) | 3.02 (0.99 to 10.09) |
|  | Delta Wave (May 1, 2021 to October 31, 2021) | 1.03 (0.37 to 2.78) |
|  | Omicron Wave (November 1, 2021 to March 28, 2022) | 0.52 (0.21 to 1.25) |
| Death | Age <60y | 1.08 (0.62 to 1.92) |
|  | Age ≥60y | 1.03 (0.27 to 4.01) |
|  | Male | 0.76 (0.37 to 1.54) |
|  | Female | 1.45 (0.63 to 3.34) |
|  | Previous TB History | 0.89 (0.21 to 4.2) |
|  | No Previous TB History | 1.18 (0.56 to 2.48) |
|  | Beta Wave (October 1, 2020 to April 30, 2021) | 4.06 (1.37 to 13.25) |
|  | Delta Wave (May 1, 2021 to October 31, 2021) | 0.77 (0.26 to 2.2) |
|  | Omicron Wave (November 1, 2021 to March 28, 2022) | 0.65 (0.28 to 1.5) |
| ICU Admission | Age <60y | 0.9 (0.24 to 2.71) |
|  | Age ≥60y | Not estimable |
|  | Male | 0.24 (0.01 to 1.37) |
|  | Female | 1.36 (0.25 to 5.58) |
|  | Previous TB History | Not estimable |
|  | No Previous TB History | 0.22 (0.01 to 1.22) |
|  | Beta Wave (October 1, 2020 to April 30, 2021) | Not estimable |
|  | Delta Wave (May 1, 2021 to October 31, 2021) | 2.12 (0.28 to 15.77) |
|  | Omicron Wave (November 1, 2021 to March 28, 2022) | 0.46 (0.02 to 3.19) |
| Mechanical Ventilation | Age <60y | 0.72 (0.09 to 3.57) |
|  | Age ≥60y | Not estimable |
|  | Male | 0.4 (0.02 to 2.65) |
|  | Female | 1.15 (0.02 to 16.05) |
|  | Previous TB History | Not estimable |
|  | No Previous TB History | 0.57 (0.03 to 3.66) |
|  | Beta Wave (October 1, 2020 to April 30, 2021) | Not estimable |
|  | Delta Wave (May 1, 2021 to October 31, 2021) | 2.43 (0.18 to 35.06) |
|  | Omicron Wave (November 1, 2021 to March 28, 2022) | Not estimable |

*Note some estimates not possible due to too few events in one group.

*The TB/COVID-19 Global Study Group (2022)^10^*

- The data provided to the study team consisted of participants within the 767 included who had known TB treatment outcomes. This comprised 118 (15%) individuals from 54 centres in 24 countries. Individuals initiated TB treatment as early as January 2019 and as late as November 2020. The distribution of countries and centres providing data are in the table below.

| **Country** | **Individuals with TB outcome** | **Centres** |
| --- | --- | --- |
| **Argentina** | 1 | 1 |
| **Belarus** | 1 | 1 |
| **Brazil** | 10 | 5 |
| **Canada** | 1 | 1 |
| **Chile** | 11 | 11 |
| **China** | 2 | 1 |
| **Colombia** | 1 | 1 |
| **Guinea** | 1 | 1 |
| **Honduras** | 2 | 2 |
| **India** | 18 | 3 |
| **Italy** | 11 | 6 |
| **Lithuania** | 5 | 1 |
| **Oman** | 8 | 2 |
| **Paraguay** | 10 | 2 |
| **Peru** | 3 | 2 |
| **Portugal** | 1 | 1 |
| **Romania** | 4 | 1 |
| **Russia** | 4 | 2 |
| **Serbia** | 2 | 1 |
| **Singapore** | 6 | 1 |
| **South Africa** | 2 | 1 |
| **Spain** | 6 | 5 |
| **United Kingdom** | 6 | 1 |
| **USA** | 2 | 1 |
| **24 countries** | **118 individuals** | **54 centres** |

- Overall, 5 participants had SARS-CoV-2 diagnosed 29 days to 180 days prior to TB treatment initiation, 52 had SARS-CoV-2 diagnosed with 28 days of TB treatment initiation, and 61 had SARS-CoV-2 diagnosed 29 days or more after TB treatment initiation (but before treatment completion).
- Data were provided to the study team stratified by time between SARS-CoV-2 diagnosis and TB, and are contained in the tables below.

**Table. Demographic, epidemiological and clinical characteristics of 118 TB/COVID- 19 cases**

Data reported as number/total number of patients for whom data are available (%)

| **Variables** | | **COVID diagnosis** | | | | | |
| --- | --- | --- | --- | --- | --- | --- | --- |
|  |  | **29-180d before TB diagnosis**  **N=5** | | **28d before to 28d after TB diagnosis**  **N=52** | | **>29d after TB diagnosis**  **N=61** | |
|  |  | **No ImmunoMod Tx**  **n=3** | **Received ImmunoMod Tx**  **n=2** | **No ImmunoMod Tx**  **n=39** | **Received ImmunoMod Tx**  **n=13** | **No ImmunoMod Tx**  **N=53** | **Received ImmunoMod Tx**  **N=8** |
| Median (IQR) age, years | | 67 (24-81) | 63.5 (2.1) | 42 (32-72) | 57 (49-78) | 44 (30-56) | 62.5 (48.5-66.5) |
| Males, n (%) | | 1/3 (33.3) | 2/2 (100) | 28/39 (71.8) | 13/13 (100) | 38/53 (71.7) | 5/8 (62.5) |
| Immigrated in the last 5 years, n (%) | | 0 (0) | 0 (0) | 3/35 (8.6) | 0 (0) | 3/50 (6.0) | 2/8 (25.0) |
| Occupation, n (%) | Unemployed | 0 (0) | ½ (50) | 8/32 (25) | 4/12 (33.3) | 25/52 (48.1) | 3/8 (37.5) |
|  | Employed | ½ (50) | ½ (50) | 13/32 (40.1) | 2/12 (16.7) | 19/52 (36.5) | 2/8 (25.0) |
|  | Retired | ½ (50) | 0 (0) | 10/32 (31.2) | 6/12 (50) | 6/52 (11.5) | 2/8 (25.0) |
|  | Student | 0 (0) | 0 (0) | 1/32 (3.1) | 0 (0) | 2/52 (3.8) | 1/8 (12.5) |
| BCG vaccinated n (%) | | 1/1 (100) | 2/2 (100) | 9/10 (90) | 8/8 (100) | 32/36 (88.9) | 7/7 (100) |
| Pregnancy, n (%) | | 2/2 (100) | - | 0 (0) | - | 0 (0) | 0 (0) |
| Alcohol abuse (≥14 drinks per week in men or ≥7 drinks per week in women), n (%) | | 1/3 (33.3) | 0 (0) | 5/36 (13.9) | 4/10 (40) | 9/51 (17.6) | 1/8 (12.5) |
| Smoking status, n (%) | No smoker | 2/3 (66.7) | ½ (50) | 20/33 (60.6) | 3/11 (27.3) | 26/44 (59.1) | 7/8 (87.5) |
|  | Current smoker | 1/3 (33.3) | 0 (0) | 10/33 (30.3) | 3/11 (27.3) | 14/44 (31.8) | 1/8 (12.5) |
|  | Former smoker | 0 (0) | ½ (50) | 3/33 (9.1) | 5/11 (45.5) | 4/44 (9.1) | 0 (0) |
| Vaping Status, n (%) | No vape | 3/3 (100) | 2/2 (100) | 22/23 (95.7) | 8/8 (100) | 32/33 (97.0) | 8/8 (100) |
|  | Current vape | 0 (0) | 0 (0) | 1/23 (4.3) | 0 (0) | 1/33 (3.0) | 0 (0) |
|  | Former vape | 0 (0) | 0 (0) | 0 (0) | 0 (0) | 0 (0) | 0 (0) |
| Intravenous Drug User (IVDU), n (%) | No drug user | 2/2 (100) | 2/2 (100) | 30/31 (96.8) | 8/10 (80) | 40/43 (93.0) | 7/8 (87.5) |
|  | Current/regular | 0 (0) | 0 (0) | 0 (0) | 2/10 (20) | 1/43 (2.3) | 0 (0) |
|  | Current/not regular | 0 (0) | 0 (0) | 0 (0) | 0 (0) | 0 (0) | 0 (0) |
|  | Former drug user | 0 (0) | 0 (0) | 1/31 (3.2) | 0 (0) | 2/43 (4.7) | 1/8 (12.5) |
| HIV positivity, n (%) | | 0 (0) | 0 (0) | 6/39 (15.4) | 2/13 (15.4) | 8/53 (15.1) | 0 (0) |
| Median (IQR) CD4 Count pre-COVID-19 infection, cells/μL | | - | - | No info | No info | 202 (118-337) | - |
| Median (IQR) CD4 Count during COVID-19 infection, cells/μL | | - | - | No info | No info | 154.5(80-229) | - |
| HIV treatment administered, n (%) | | - | - | 1/1 (100) | 1/1 (100) | 5/8 (62.5) | - |
| COPD, n (%) | | 0 (0) | 0 (0) | 5/39 (12.8) | 1/13 (7.7) | 3/53 (5.7) | 1/8 (12.5) |
| Diabetes Mellitus (DM), n (%) | | 1 (33.3) | 2/2 (100) | 7/39 (17.9) | 4/13 (30.8) | 8/53 (15.1) | 3/8 (37.5) |
| Uncontrolled DM (HbA1c ≥9%), n (%) | | 0 (0) | 0 (0) | 1/7 (14.2) | 0 (0) | 2/8 (25.0) | 1/3 (33.3) |
| Poorly controlled DM (HbA1c 7 – 9%), n (%) | | 0 (0) | 0 (0) | 3/7 (42.9) | 1/1 (100) | 1/8 (12.5) | 1/3 (33.3) |
| Well controlled DM (HbA1c <7%), n (%) | | 0 (0) | 0 (0) | 0 (0) | 0 (0) | 4/8 (50.0) | 0 (0) |
| Unknown DM control, n (%) | | 1/1 (100) | 2/2 (100) | 3/7 (42.9) | 0 (0) | 1/8 (12.5) | 1/3 (33.3) |
| Renal Failure, n (%) | | 0 (0) | 0 (0) | 3/39 (7.7) | 1/13 (7.7) | 1/53 (1.9) | 1/8 (12.5) |
| Dialysis, n (%) | | - | - | 0 (0) | 0 (0) | 0 (0) | 0 (0) |
| Liver disease, n (%) | | 1/3 (33.3) | ½ (50) | 1/39 (2.6) | 1/13 (7.7) | 3/53 (5.7) | 3/8 (37.5) |
| **TB outcomes** | | | | | | | |
| **SUCCESS, n (%)** | | 1/3 (33.3) | 0 (0) | 14/39 (35.9) | 2/13 (15.4) | 38/53 (71.7) | 1/8 (12.5) |
| **FAILURE, n (%)** | | 0 (0) | 0 (0) | 0 (0) | 0 (0) | 0 (0) | 0 (0) |
| **DEATH, n(%)** | | 2/3 (66.7) | 2/2 (100) | 19/39 (48.7) | 10/13 (76.9) | 11/53 (20.8) | 6/8 (75.0) |
| *Death due to TB, n(%)* | | *0 (0)* | *0 (0)* | *0 (0)* | *0 (0)* | *0 (0)* | *0 (0)* |
| *Death due to TB+COVID, n(%)* | | *2/2 (100)* | *2/2 (100)* | *13/19 (68.4)* | *7/13 (53.8)* | *5/11 (45.5)* | *2/6 (33.3)* |
| *Death due to COVID, n(%)* | | *0 (0)* | *0 (0)* | *2/19 (10.5)* | *3/13 (23.1)* | *2/11 (18.2)* | *4/6 (66.7)* |
| *Death due to OTHER, n(%)* | | *0 (0)* | *0 (0)* | *4/19 (21.1)* | *0 (0)* | *4/11 (36.4)* | *0 (0)* |
| **LOSS TO FU, n (%)** | | 0 (0) | 0 (0) | 6/39 (15.4) | 1/13 (7.7) | 4/53 (7.5) | 1/8 (12.5) |
| **COVID-19 outcomes** | | | | | | | |
| **NEVER HOSP, n(%)** | Symp Resolved | 1/3 (33.3) | 0 (0) | 2/39 (5.1) | 0 (0) | 20/53 (37.7) | 1/8 (12.5) |
|  | Symp Unresolved | 0 (0) | 0 (0) | 1/39 (2.6) | 0 (0) | 1/53 (1.9) | 0 (0) |
| **DISCHARGED, n(%)** | Symp Resolved | 0 (0) | 0 (0) | 16/39 (41) | 3/13 (23.1) | 20/53 (37.7) | 0 (0) |
|  | Symp Unresolved | 0 (0) | 0 (0) | 0 (0) | 0 (0) | 0 (0) | 0 (0) |
| **STILL HOSP, n(%)** | Symp Resolved | 0 (0) | 0 (0) | 0 (0) | 0 (0) | 0 (0) | 0 (0) |
|  | Symp Unresolved | 0 (0) | 0 (0) | 1/39 (2.6) | 0 (0) | 1/53 (1.9) | 1/8 (12.5) |
| **DEATH, n(%)** | | 2/3 (66.7) | 2/2 (100) | 19/39 (48.7) | 10/13 (76.9) | 11/53 (20.8) | 6/8 (75.0) |
| *Death due to TB, n(%)* | | *0 (0)* | *0 (0)* | *0 (0)* | *0 (0)* | *0 (0)* | *0 (0)* |
| *Death due to TB+COVID, n(%)* | | *2/2 (66.7)* | *2/2 (100)* | *13/19 (68.4)* | *7/13 (53.8)* | *5/11 (45.5)* | *2/6 (33.3)* |
| *Death due to COVID, n(%)* | | *0 (0)* | *0 (0)* | *2/19 (10.5)* | *3/13 (23.1)* | *2/11 (18.2)* | *4/6 (66.7)* |
| *Death due to OTHER, n(%)* | | *0 (0)* | *0 (0)* | *4/19 (21.1)* | *0 (0)* | *4/11 (36.4)* | *0 (0)* |
| **Unknown, n (%)** | | 0 (0) | 0 (0) | 0 (0) | 0 (0) | 0 (0) | 0 (0) |

**Table. Descriptive analysis of TB in the TB/COVID-19 cohort**

|  | | **COVID diagnosis** | | | | | |
| --- | --- | --- | --- | --- | --- | --- | --- |
|  | | **29-180d before TB diagnosis**  **N=5** | | **28d before to 28d after TB diagnosis N=52** | | **>29d after TB diagnosis**  **N=61** | |
| **TUBERCULOSIS** | | **No ImmunoMod Tx**  **n=3** | **Received ImmunoMod Tx**  **n=2** | **No ImmunoMod Tx**  **n=39** | **Received ImmunoMod Tx**  **n=13** | **No ImmunoMod Tx**  **N=53** | **Received ImmunoMod Tx**  **N=8** |
| TB Form, n (%) | Failure | 0 (0) | 0 (0) | 0 (0) | 0 (0) | 1/53 (1.9) | 0 (0) |
|  | Relapsed | 0 (0) | 0 (0) | 0 (0) | 5/13 (38.5) | 10/53 (18.9) | 0 (0) |
|  | Lost to Follow up | 1/3 (33.3) | 0 (0) | 0 (0) | 0 (0) | 4/53 (7.5) | 2/7 (28.6) |
|  | New case | 2/3 (66.7) | 2/2 (100) | 39/39 (100) | 8/13 (61.5) | 38/53 (71.7) | 5/7 (71.4) |
| TB laboratory confirmation, n (%) | | 3/3 (100) | 2/2 (100) | 23/38 (60.5) | 11/13 (84.6) | 47/53 (88.7) | 7/8 (87.5) |
| **Site** | | | | | | | |
| Pulmonary TB only, n (%) | | 2/3 (66.7) | 2/2 (100) | 28/39 (71.8) | 11/13 (84.6) | 37/53 (69.8) | 5/8 (62.5) |
| Pulmonary + Extra-Pulmonary TB, n (%) | | 1/3 (33.3) | 0 (0) | 4/39 (10.3) | 1/13 (7.7) | 7/53 (13.2) | 1/8 (12.5) |
| Extra-Pulmonary TB only, n (%) | | 0 (0) | 0 (0) | 7/39 (17.9) | 1/13 (7.7) | 9/53 (17.0) | 2/8 (25.0) |
| Site Extra-Pulm TB, n (%) | Pleural TB | 1/1 (100) | - | 6/11 (54.5) | 0 (0) | 4/16 (25.0) | 0 (0) |
|  | TB Lymphadenitis | 0 (0) | - | 2/11 (18.2) | 0 (0) | 6/16 (37.5) | 1/3 (33.3) |
|  | Multiple locations | 0 (0) | - | 1/11 (9.1) | 0 (0) | 2/16 (12.5) | 0 (0) |
|  | Central Nervous System | 0 (0) | - | 1/11 (9.1) | ½ (50) | 2/16 (12.5) | 0 (0) |
|  | Other | 0 (0) | - | 1/11 (9.1) | ½ (50) | 0 (0) | 0 (0) |
|  | Bone TB | 0 (0) | - | 0 (0) | 0 (0) | 1/16 (6.3) | 1/3 (33.3) |
|  | Gastrointestinal TB | 0 (0) | - | 0 (0) | 0 (0) | 0 (0) | 1/3 (33.3) |
|  | TB Peritonitis, | 0 (0) | - | 0 (0) | 0 (0) | 0 (0) | 0 (0) |
|  | Genitourinary TB | 0 (0) | - | 0 (0) | 0 (0) | 1/16 (6.3) | 0 (0) |
|  | TB Pericarditis | 0 (0) | - | 0 (0) | 0 (0) | 0 (0) | 0 (0) |
|  | Unknown | 0 (0) | - | 0 (0) | 0 (0) | 0 (0) | 0 (0) |
| **Radiology at TB diagnosis** | | | | | | | |
| Bilateral pulmonary cavitary lesion, n (%) | | 0 (0) | ½ (50) | 3/33 (9.1) | 2/13 | 8/46 (17.4) | 2/8 (25.0) |
| Bilateral pulmonary cavitary lesion + other, n (%) | | 0 (0) | 0 (0) | 0 (0) | 0 (0) | 0 (0) | 0 (0) |
| Unilateral pulmonary cavitary lesion, n (%) | | 0 (0) | 0 (0) | 7/33 (21.2) | 1/13 (7.7) | 8/46 (17.4) | 2/8 (25.0) |
| Unilateral pulmonary cavitary lesion + other, n (%) | | 0 (0) | 0 (0) | 0 (0) | 1/13 (7.7) | 0 (0) | 0 (0) |
| Bilateral pulmonary infiltrate (no cavities), n (%) | | 2/2 (100) | 0 (0) | 8/33 (24.2) | 0 (0) | 5/46 (10.9) | 0 (0) |
| Bilateral pulmonary infiltrate (no cavities)+ other, n (%) | | 0 (0) | 0 (0) | 0 (0) | 7/13 (53.8) | 0 (0) | 0 (0) |
| Unilateral pulmonary infiltrate (no cavities), n (%) | | 0 (0) | 0 (0) | 6/33 (18.2) | 1/13 (7.7) | 11/46 (23.9) | 2/8 (25.0) |
| Unilateral pulmonary infiltrate (no cavities)+ other, n (%) | | 0 (0) | 0 (0) | 0 (0) | 0 (0) | 0 (0) | 0 (0) |
| Other lesions, n (%) | | 0 (0) | ½ (50) | 9/33 (27.3) | 1/13 (7.7) | 13/46 (28.3) | 2/8 (25.0) |
| Not done, n (%) | | 0 (0) | 0 (0) | 0 (0) | 0 (0) | 1/46 (2.2) | 0 (0) |
| **Lung function tests at TB diagnosis** | | | | | | | |
| Lung function tests done at TB diagnosis, n (%) | | 2/3 (66.7) | ½ (50) | 20/31 (64.5) | 4/11 (36.4) | 14/48 (29.2) | 2/8 (25.0) |
| Median (IQR) sO2, % | | 97.5 (97-98) | No info | 97 (93-98) | 96,5 (93-99) | 97 (96-98) | No info |
| Median (IQR) FiO2, % | | 21 (21-21) | No info | 21 (21-29) | 28 (21-54) | 21 (21-21) | No info |
| Median (IQR) pO2, mmHg | | No info | No info | 89 (67-147) | 71 (66-224) | No info | No info |
| Median (IQR) pCO2, mmHg | | No info | No info | 29 (24-46) | 32 (27-42) | No info | No info |
| Median (IQR) pH | | No info | No info | 7.5 (7.4-7.5) | 7.4 (7.4-7.5) | No info | No info |
| **Microbiology** | | | | | | | |
| TB microbiology done (one or more tests), n (%) | | 2/2 (100) | 2/2 (100) | 31/32 (96.9) | 12/13 (92.3) | 51/53 (96.2) | 7/8 (87.5) |
| Solid culture, n (%) | | ½ (50) | ½ (50) | 20/31 (64.5) | 8/12 (66.7) | 29/51 (56.9) | 1/7 (14.3) |
| Gene Xpert, n (%) | | 2/2 (100) | ½ (50) | 20/31 (64.5) | 6/12 (50) | 32/51 (62.7) | 6/7 (85.7) |
| Liquid culture, n (%) | | ½ (50) | 2/2 (100) | 23/31 (74.2) | 5/12 (41.7) | 26/51 (51.0) | 2/7 (28.6) |
| First line LPA, n (%) | | 0 (0) | ½ (50) | 0 (0) | 2/12 (16.7) | 10/51 (19.6) | 1/7 (14.3) |
| Second line LPA, n (%) | | 0 (0) | 0 (0) | 0 (0) | 0 (0) | 3/51 (5.9) | 1/7 (14.3) |
| **Drug resistance pattern at TB diagnosis** | | | | | | | |
| Pan susceptible-TB, n (%) | | 3 (100) | 2/2 (100) | 23/27 (85.2) | 6/7 (85.7) | 36/45 (80.0) | 4/6 (66.7) |
| Drug resistant-TB, n (%) | | 0 (0) | 0 (0) | 4/27 (14.8) | 1/7 (14.3) | 9/45 (20.0) | 2/6 (33.3) |
| **Hospitalization** | | | | | | | |
| Hospitalization during anti-TB treatment, n (%) | | 2/3 (66.7) | 2/2 (100) | 34/36 (94.4) | 13/13 (100) | 21/47 (44.7) | 4/8 (50) |
| Median (IQR) duration of hospitalization, days | | info available for 1 pt only: 5d | 25 (5-46) | 20 (10-40) | 14 (6-27) | 87 (11-203) | 44 (29-123) |
| **Interim TB outcomes** | | | | | | | |
| Median (IQR) days sputum culture conversion | | info available for 1 pt only; 14d | No info | 30 (27-60) | No info | 58.5 (35-88) | info available for 1 pt only; 144d |

**Table. Descriptive analysis of COVID-19 in the TB/COVID-19 cohort**

|  | | | **COVID diagnosis** | | | | | | |
| --- | --- | --- | --- | --- | --- | --- | --- | --- | --- |
|  | | | | **29-180d before TB diagnosis**  **N=5** | | **28d before to 28d after TB diagnosis N=52** | | **>29d after TB diagnosis**  **N=61** | |
| **COVID-19** | | | | **No ImmunoMod Tx**  **n=3** | **Received ImmunoMod Tx**  **n=2** | **No ImmunoMod Tx**  **n=39** | **Received ImmunoMod Tx**  **n=13** | **No ImmunoMod Tx**  **N=53** | **Received ImmunoMod Tx**  **N=8** |
| SARS-CoV-2 laboratory confirmation, n (%) | | | | 3/3 (100) | 2/2 (100) | 37/39 (94.9) | 13/13 (100) | 51/53 (96.2) | 8/8 (100) |
| **Signs and symptoms** | | | | | | | | | |
| COVID-19 signs and symptoms (one or more symptoms), n(%) | | | | 2/2 (100) | 2/2 (100) | 33/38 (86.8) | 11/13 (84.6) | 44/50 (88.0) | 7/8 (87.5) |
| Fever, n (%) | | | | ½ (50) | ½ (50) | 26/33 (78.8) | 9/11 (81.8) | 29/44 (65.9) | 5/7 (71.4) |
| Dry cough, n (%) | | | | 0 (0) | 2/2 (100) | 22/33 (66.7) | 5/11 (45.5) | 20/44 (45.5) | 6/7 (85.7) |
| Shortness of breath, n (%) | | | | 0 (0) | ½ (50) | 15/33 (45.5) | 5/11 (45.5) | 9/44 (20.5) | 3/7 (42.9) |
| Headache, n (%) | | | | 0 (0) | 0 (0) | 3/33 (9.1) | 3/11 (27.3) | 8/44 (18.2) | 1/7 (14.3) |
| Tiredness, n (%) | | | | 0 (0) | ½ (50) | 6/33 (18.2) | 2/11 (18.2) | 8/44 (18.2) | 1/7 (14.3) |
| Sore throat, n (%) | | | | 0 (0) | 0 (0) | 3/33 (9.1) | 0 (0) | 7/44 (15.9) | 1/7 (14.3) |
| Malaise, n (%) | | | | 0 (0) | 0 (0) | 7/33 (21.2) | 1/11 (9.1) | 8/44 (18.2) | 2/7 (28.6) |
| Chest pain, n (%) | | | | 0 (0) | 0 (0) | 4/33 (12.1) | 4/11 (36.4) | 1/44 (2.3) | 0 (0) |
| Myalgia, n (%) | | | | 0 (0) | 0 (0) | 5/33 (15.2) | 0 (0) | 6/44 (13.6) | 2/7 (28.6) |
| Nasal congestion, n (%) | | | | 0 (0) | 0 (0) | 1/33 (3) | 1/11 (9.1) | 4/44 (9.1) | 2/7 (28.6) |
| Taste disorders, n (%) | | | | 0 (0) | 0 (0) | 2/33 (6.1) | 2/11 (18.2) | 2/44 (4.5) | 0 (0) |
| Diarrhoea, n (%) | | | | 0 (0) | 0 (0) | 3/33 (9.1) | 0 (0) | 0 (0) | 1/7 (14.3) |
| Olfactory disorders, n (%) | | | | ½ (50) | 0 (0) | 3/33 (9.1) | 0 (0) | 1/44 (2.3) | 0 (0) |
| Vomiting/nausea, n (%) | | | | 0 (0) | 0 (0) | 4/33 (12.1) | 2/11 (18.2) | 6/44 (13.6) | 3/7 (42.9) |
| Arthralgia, n (%) | | | | 0 (0) | 0 (0) | 2/33 (6.1) | 1/11 (9.1) | 2/44 (4.5) | 0 (0) |
| Abdominal pain, n (%) | | | | 0 (0) | 0 (0) | 1/33 (3) | 0 (0) | 3/44 (6.8) | 1/7 (14.3) |
| Irritability/confusion, n (%) | | | | 0 (0) | 0 (0) | 3/33 (9.1) | 1/11 (9.1) | 3/44 (6.8) | 1/7 (14.3) |
| Other symptoms (loss of appetite rhinorrea, difficulty of breathing, hemoptisys, conjunctivitis among others), n (%) | | | | 0 (0) | 0 (0) | 10/33 (30.3) | 1/11 (9.1) | 3/44 (6.8) | 1/7 (14.3) |
| **Radiology at diagnosis** | | | | | | | | | |
| CT SCAN only, n (%) | | | | 0 (0) | 0 (0) | 4/33 (12.1) | 1/13 (7.7) | 5/48 (10.4) | 1/8 (12.5) |
| Chest X ray only, n (%) | | | | 0 (0) | ½ (50) | 15/33 (45.5) | 4/13 (30.8) | 20/48 (41.7) | 5/8 (62.5) |
| CT SCAN and Chest X ray, n (%) | | | | 0 (0) | ½ (50) | 12/33 (36.4) | 7/13 (53.8) | 2/48 (4.2) | 1/8 (12.5) |
| Radiology not done, n (%) | | | | 3/3 (100) | 0 (0) | 2/33 (6.1) | 1/13 (7.7) | 21/48 (43.8) | 1/8 (12.5) |
| CT scan findings, n (%) | Typical Ground opacity/opacities, bilateral | | | - | 1/1 (100) | 6/16 (37.5) | 3/8 (37.5) | 2/7 (28.6) | 2/2 (100) |
|  | Typical Ground opacity/opacities, unilateral | | | - | 0 (0) | 1/16 (6.3) | 1/8 (12.5) | 4/7 (57.1) | 0 (0) |
|  | Atypical Ground opacity/opacities | | | - | 0 (0) | 5/16 (31.3) | 3/8 (37.5) | 1/7 (14.3) | 0 (0) |
|  | Typical Ground opacity bilateral and atypical ones | | | - | 0 (0) | 3/16 (18.8) | 1/8 (12.5) | 0 (0) | 0 (0) |
|  | No COVID-19 lesion(s) (no opacity) | | | - | 0 (0) | 1/16 (6.3) | 0 (0) | 0 (0) | 0 (0) |
| **Lung function tests at COVID-19 diagnosis** | | | | | | | | | |
| Lung function tests at COVID-19 diagnosis, n (%) | | | | 0 (0) | ½ (50) | 21/29 (72.4) | 8/11 (72.7) | 29/48 (60.4) | 7/8 (87.5) |
| Median (IQR) sO2, % | | | | - | 91 (1 pt) | 97 (95-98) | 96 (89-97) | 96 (95-98) | 80 (75-87) |
| Median (IQR) FiO2, % | | | | - | No info | 21 (21-26) | 36 (35-50) | 21 (21-21) | 21 (21-21) |
| Median (IQR) pO2, mmHg | | | | - | No info | 102 (70-154) | 106 (66-159) | No info | 51 (48-62) |
| Median (IQR) pCO2, mmHg | | | | - | No info | 32 (29-40) | 37.5 (32.8-46.2) | No info | 29 (27-69) |
| Median (IQR) pH | | | | - | No info | 7.4 (7.4-7.5) | 7.4 (7.3-7.4) | No info | 7.2 (7.2-7.3) |
| **Ventilation and oxygen therapy** | | | | | | | | | |
| No ventilation, n (%) | | | | ½ (50) | ½ (50) | 22/32 (68.8) | 9/13 (69.2) | 43/48 (89.6) | 2/8 (25.0) |
| Supplemental oxygen during Covid-19, n(%) | | | | ½ (50) | 2/2 (100) | 15/32 (46.9) | 8/11 (72.7) | 8/49 (16.3) | 8/8 (100) |
| Mechanical ventilation during Covid-19, n (%) | | Non-invasive | | ½ (50) | 0 (0) | 6/32 (18.8) | 1/13 (7.7) | 3/48 (6.3) | 1/8 (12.5) |
|  |  | Invasive | | 0 (0) | ½ (50) | 4/32 (12.5) | 3/13 (23.1) | 2/48 (4.2) | 5/8 (62.5) |
| **Hospitalization** | | | | | | | | | |
| Hospitalization due to COVID-19, n (%) | | | | 2/3 (66.7) | 2/2 (100) | 34/39 (87.2) | 9/13 (69.2) | 26/53 (49.1) | 7/8 (87.5) |
| Median (IQR)/Mean (SD) duration of hospitalization, days | | | | info available for 1 pt only: 7d | 40 (39-41) | 14 (9.5-25.5) | 14 (6-25) | 14.5 (7-35) | 5 (3-15) |
| Concomitant hospitalization due to TB-COVID-19 co-infection, n (%) | | | | 2/3 (66.7) | ½ (50) | 32/39 (82.1) | 8/13 (61.5) | 12/52 (23.1) | 3/8 (37.5) |
| Median (IQR) duration of concomitant hospitalization, days | | | | info available for 1 pt only: 5d | 8 (1 pt) | 18 (10-26) | 11.5 (6-19) | 62 (2-209) | 5 (1-33) |
| **PCR conversion rates** | | | | | | | | | |
| PCR conversion, n (%) | | | | ½ (50) | 1/1 (100) | 12/25 (48.0) | 3/9 (33.3) | 23/40 (57.5) | 1/7 (14.3) |
| Median (IQR) from start treatment to PCR conversion, days | | | | No info | 39 (1 pt) | 18.5 (10-31) | 19 (9-22) | 13.5 (10-23) | 73 (1 pt) |
| **Treatment** | | | | | | | | | |
| Treatment for COVID-19 **(**one or more drugs), n (%) | | | | 1/3 (33.3) | 2/2 (100) | 17/31 (54.8) | 13/13 (100) | 24/47 (51.1) | 8/8 (100) |
| **Antivirals** | | | | | | | | | |
| Lopinavir/Ritonavir, n (%) | | | | 0 (0) | 0 (0) | 3/17 (17.6) | 2/13 (15.4) | 0 (0) | 2/8 (25.0) |
| Darunavir/Cobicistat or Darunavir/Ritonavir, n (%) | | | | 0 (0) | ½ (50) | 0 (0) | 1/13 (7.7) | 1/24 (4.2) | 0 (0) |
| Favipiravir, n (%) | | | | 0 (0) | 0 (0) | 0 (0) | 0 (0) | 2/24 (8.3) | 0 (0) |
| Remdesivir, n (%) | | | | 0 (0) | 0 (0) | 0 (0) | 0 (0) | 2/24 (8.3) | 0 (0) |
| Other antivirals, n (%) | | | | 0 (0) | 0 (0) | 0 (0) | 1/13 (7.7) | 0 (0) | 0 (0) |
| **Anticoagulants** | | | | 0 (0) | 0 (0) | 0 (0) | 0 (0) | 0 (0) | 0 (0) |
| Enoxaparin, n (%) | | | | 0 (0) | 0 (0) | 0 (0) | 5/13 (38.5) | 4/24 (16.7) | 2/8 (25.0) |
| Other therapeutic anticoagulants, n (%) | | | | 0 (0) | 0 (0) | 0 (0) | 1/13 (7.7) | 0 (0) | 0 (0) |
| **Immunomodulators** | | | | | | | | | |
| Glucocorticoids (methylprednisolone, betamethasone, ciclesonide , other glucocorticoids), n (%) | | | | 0 (0) | 2/2 (100) | 0 (0) | 13/13 (100) | 0 (0) | 8/8 (100) |
| Intravenous immunoglobulin (IVIG), n (%) | | | | 0 (0) | 0 (0) | 0 (0) | 1/13 (7.7) | 0 (0) | 0 (0) |
| Interleukin (IL)-6 inhibitors, n (%) | | | | 0 (0) | 0 (0) | 0 (0) | 0 (0) | 0 (0) | 0 (0) |
| Bevacizumab (antibody against VEGF-A), n (%) | | | | 0 (0) | 0 (0) | 0 (0) | 0 (0) | 0 (0) | 0 (0) |
| **Miscellaneous** | | | | | | | | | |
| Azithromycin, n (%) | | | | 0 (0) | ½ (50) | 9/17 (52.9) | 7/13 (53.8) | 14/24 (58.3) | 5/8 (62.5) |
| Hydroxychloroquine, n (%) | | | | 1/1 (100) | ½ (50) | 9/17 (52.9) | 5/13 (38.5) | 17/24 (70.8) | 6/8 (75.0) |
| N-acetyl-cysteine, n (%) | | | | 0 (0) | 0 (0) | 0 (0) | 1/13 (7.7) | 0 (0) | 2/8 (25.0) |
| Plasma from recovered patients, n (%) | | | | 0 (0) | 0 (0) | 0 (0) | 0 (0) | 0 (0) | 0 (0) |
| Interferon, n (%) | | | | 0 (0) | 0 (0) | 0 (0) | 0 (0) | 0 (0) | 0 (0) |
| Other non-steroid anti-inflammatory drugs, n (%) | | | | 0 (0) | 0 (0) | 0 (0) | 0 (0) | 0 (0) | 0 (0) |
| **Number of comorbidities** | | | | | | | | | |
| 0 | | | | 1/3 (33.3) | 0 (0) | 16/39 (41) | 3/13 (23.1) | 28/53 (52.8) | 2/8 (25.0) |
| 1 | | | | 0 (0) | 0 (0) | 10/39 (25.6) | 7/13 (53.8) | 18/53 (34.0) | 1/8 (12.5) |
| 2 | | | | 1/3 (33.3) | ½ (50) | 5/39 (12.8) | 2/13 (15.4) | 3/53 (5.7) | 3/8 (37.5) |
| 3 | | | | 1/3 (33.3) | ½ (50) | 4/39 (10.3) | 0 (0) | 3/53 (5.7) | 0 (0) |
| 4 | | | | 0 (0) | 0 (0) | 0 (0) | 1/13 (7.7) | 1/53 (1.9) | 1/8 (12.5) |
| 5 | | | | 0 (0) | 0 (0) | 0 (0) | 0 (0) | 0 (0) | 0 (0) |
| 6 | | | | 0 (0) | 0 (0) | 3/39 (7.7) | 0 (0) | 0 (0) | 1/8 (12.5) |
| 7 | | | | 0 (0) | 0 (0) | 1/39 (2.6) | 0 (0) | 0 (0) | 0 (0) |
| 8 | | | | 0 (0) | 0 (0) | 0 (0) | 0 (0) | 0 (0) | 0 (0) |

**Table. Characteristics of the patients with COVID diagnosis from 28 days before to 28 days after TB diagnosis with and without immunomodulating treatment**

| **Characteristic** | **COVID diagnosis 28d before to 28d after TB diagnosis**  **N=52** | | **p-value** |
| --- | --- | --- | --- |
|  | **No ImmunoMod Tx**  **n=39** | **Received ImmunoMod Tx**  **n=13** |  |
| Median (IQR) age, years | 42 (32-72) | 57 (49-78) | 0.10 |
| Age ≥65 years, n (%) | 12/39 (30.8) | 5/13 (38.5) | 0.74 |
| Males, n (%) | 28/39 (71.8) | 13/13 (100) | 0.047 |
| Non-European, n (%) | 13/39 (33.3) | 4/13 (30.8) | 0.87 |
| ≥1 comorbidity, n (%) | 23/39 (59.0) | 10/13 (76.9) | 0.33 |
| ≥2 comorbidities, n (%) | 13/39 (33.3) | 3/13 (23.1) | 0.73 |
| Median (IQR) no. of comorbidities | 1 (0-2) | 1 (0.5-1.5) | 0.59 |
| Diabetes Mellitus, n (%) | 7/39 (17.9) | 4/13 (30.8) | 0.44 |
| Cardiovascular Disease, n (%) | 10/39 (25.6) | 2/13 (15.4) | 0.706 |
| Chronic Resp. Disease, n (%) | 9/39 (23.1) | 1/13 (7.7) | 0.42 |
| HIV, n (%) | 6/39 (15.4) | 2/13 (15.4) | 1.0 |
| Chronic Liver Disease, n (%) | 1/39 (2.6) | 1/13 (7.7) | 0.44 |
| Chronic Renal Disease, n (%) | 3/39 (7.7) | 1/13 (7.7) | 1.0 |
| Invasive ventilation, n (%) | 4/32 (12.5) | 3/13 (23.1) | 0.39 |
| Hospitalization due to COVID-19, n (%) | 34/39 (87.2) | 9/13 (69.2) | 0.20 |
| Require oxygen, n(%)  (of those hospitalized) | 14/29 (48.3) | 6/9 (66.7) | 0.45 |
| Median (IQR) duration of hospitalization due to COVID-19, days | 14 (9.5-25.5) | 14 (6-25) | 0.45 |
| Concomitant hospitalization due to TB-COVID-19 co-infection, n (%) | 32/39 (82.1) | 8/13 (61.5) | 0.15 |
| Median (IQR) duration of concomitant hospitalization, days | 18 (10-26) | 11.5 (6-19) | 0.19 |
| TB Treatment Success, n(%) | 14/39 (35.9) | 2/13 (15.4) | 0.30 |
| Median (IQR) Time to culture conversion, days | 30 (27-60) | No info | - |
| Mortality, n(%) | 19/39 (48.7) | 10/13 (76.9) | 0.11 |
| Remain in hospital, n(%) | 1/39 (2.6) | 0 (0) | 1.0 |

**Table. Characteristics of the patients with COVID diagnosis 29-180 days after TB diagnosis with and without immunomodulating treatment**

| **Characteristic** | **COVID diagnosis >29d after TB diagnosis**  **N=61** | | **p-value** |
| --- | --- | --- | --- |
|  | **No ImmunoMod Tx**  **N=53** | **Received ImmunoMod Tx**  **N=8** |  |
| Median (IQR) age, years | 44 (30-56) | 62.5 (48.5-66.5) | 0.026 |
| Age ≥65 years, n (%) | 5/53 (9.4) | 3/8 (37.5) | 0.06 |
| Males, n (%) | 38/53 (71.7) | 5/8 (62.5) | 0.68 |
| Non-European, n (%) | 35/53 (66.0) | 6/8 (75.0) | 0.61 |
| ≥1 comorbidity, n (%) | 25/53 (47.2) | 6/8 (75.0) | 0.25 |
| ≥2 comorbidities, n (%) | 7/53 (13.2) | 5/8 (62.5) | 0.005 |
| Median (IQR) no. of comorbidities | 0.0 (0.0-1.0) | 2.0 (0.3-3.5) | 0.019 |
| Diabetes Mellitus, n (%) | 8/53 (15.1) | 3/8 (37.5) | 0.15 |
| Cardiovascular Disease, n (%) | 7/53 (13.2) | 4/8 (50.0) | 0.029 |
| Chronic Resp. Disease, n (%) | 5/53 (9.4) | 2/8 (25.0) | 0.23 |
| HIV, n (%) | 8/53 (15.1) | 0.0 (0.0) | 0.59 |
| Chronic Liver Disease, n (%) | 3/53 (5.7) | 3/8 (37.5) | 0.025 |
| Chronic Renal Disease, n (%) | 1/53 (1.9) | 1/8 (12.5) | 0.25 |
| Invasive ventilation, n (%) | 2/48 (4.2) | 5/8 (62.5) | <0.0001 |
| Hospitalization due to COVID-19, n (%) | 26/53 (49.1) | 7/8 (87.5) | 0.06 |
| Require oxygen, n(%)  (of those hospitalized) | 8/49 (16.3) | 8/8 (100.0) | <0.0001 |
| Median (IQR) duration of hospitalization due to COVID-19, days | 14.5 (7-35) | 5.0 (3.0-15.0) | 0.11 |
| Concomitant hospitalization due to TB-COVID-19 co-infection, n (%) | 12/52 (23.1) | 3/8 (37.5) | 0.4 |
| Median (IQR) duration of concomitant hospitalization, days | 62.0 (2.0-209.0) | 5.0 (1.0-33.0) | 0.39 |
| TB Treatment Success, n(%) | 38/53 (71.7) | 1/8 (12.5) | 0.002 |
| Median (IQR) Time to culture conversion, days | 58.5 (35.0-88.0) | info available for 1 pt only; 144d | 0.11 |
| Mortality, n(%) | 11/53 (20.8) | 6/8 (75.0) | 0.004 |
| Remain in hospital, n(%) | 1/53 (1.9) | 1/8 (12.5) | 0.25 |

*Davies (2022)^11^*

- The data provided to the study team are an expanded cohort of the published study. This cohort includes all patients with current, active tuberculosis who were diagnosed with COVID-19 during the Beta Wave (10 November 2020 to 22 January 2021), Delta Wave (10 June 2021 to 7 September 2021), and Omicron Wave (30 November 2021 to 10 January 2022), with waves defined as the start and end date where 7 day moving average of cases when each variant was dominant were ≥30 per 100,000 population.
- Data on SARS-CoV-2 vaccination only includes those with full vaccination (i.e., >28 days after 1 dose of Janssen vaccine or >14 days after 2 doses of Pfizer vaccine).
- Clinical guidelines state that steroid treatment (e.g., with dexamethasone) be reserved for patients requiring supplemental oxygen or mechanical ventilation (i.e., at a minimum severe COVID-19 disease)—see: <https://www.nicd.ac.za/diseases-a-z-index/disease-index-covid-19/covid-19-guidelines/clinical-management-of-suspected-or-confirmed-covid-19-disease/>. However, data on oxygen supplementation is largely incomplete in this dataset. Since steroids are reserved for such patients, there is good confidence that all who received steroids met eligibility criteria, however the population without steroids may be clinically different (not eligible for steroids or contraindicated). In addition, there is some uncertainty if patients coded as not receiving steroids in fact did not receive them, as sometimes steroids are given without prescription using ward stock, which is not captured in electronic databases.
- To try and correct for missing oxygen supplementation information, we restricted the population to individuals surviving at least 2 days from admission, as this is the average time it takes to initiate patients on steroid treatment in this context. However, the authors do not have reliable timing of steroid initiation, which inhibits our ability to do time-dependent analyses. Rather, included participants are dichotomously grouped.
- The entire shared dataset consists of 633 patients—143 receiving steroids and 490 not receiving steroids. No patients received other immunomodulators. There were 29% of patients who had their SARS-CoV-2 diagnosis prior to presentation to the hospital; 71% were diagnosed on presentation to emergency care.
- Data on ICU admission or mechanical ventilation were not available. Only discharge status (alive or died) was available.
- Characteristics of the included patients are in the table below:

|  | **No Steroids** | **Steroids** | **Total** |
| --- | --- | --- | --- |
| N | 490 | 143 | 633 |
| Median (IQR) Age, y | 40 (32; 51) | 41 (33; 53) | 40 (32; 52) |
| Age 20-39y | 236 (48.2%) | 65 (45.5%) | 301 (47.6%) |
| Age 40-59y | 195 (39.8%) | 57 (39.9%) | 252 (39.8%) |
| Age 60+ y | 59 (12.0%) | 21 (14.7%) | 80 (12.6%) |
| Female Sex | 252 (51.4%) | 67 (46.9%) | 319 (50.4%) |
| PLHIV | 294 (60.0%) | 96 (67.0%) | 390 (61.1%) |
| If PLHIV, known on ART | Data not available | Data not available | Data not available |
| Previous TB Disease | 211 (43.1%) | 7063 (44.1%) | 274 (43.3%) |
| Previous SARS-CoV-2 Diagnosis | 1 (0.2%) | 0 (0%) | 1 (0.2%) |
| From Wave 2 (Beta) | 141 (28.8%) | 40 (28.0%) | 181 (28.6%) |
| From Wave 3 (Delta) | 180 (36.7%) | 62 (43.3%) | 242 (38.2%) |
| From Wave 4 (Omicron) | 169 (34.5%) | 41 (28.7%) | 210 (33.2%) |
| Fully Vaccinated (>28d post 1 dose of Janssen or >14d post 2^nd^ Pfizer dose) | 20 (4.1%) | 6 (4.2%) | 26 (4.1%) |
| Received Supplemental O_2_ | Data not available | Data not available | Data not available |
| Median (IQR) Length of Stay, days | 9 (5 to 24) | 17 (7 to 44) | 10 (5 to 28) |
| **Discharge Status** |  |  |  |
| Alive | 382 (78.0%) | 125 (87.4%) | 507 (80.1%) |
| Died | 108 (22.0%) | 18 (12.6%) | 126 (19.9%) |

- Beginning with day 3 in hospital to date of event of interest (death) or censor (alive at discharge), the authors estimated adjusted hazard ratios for death up to 60 days after admission, adjusting for the factors of: age group (20-39y, 40-59y, and 60y or older), sex, HIV (no data on ART use was available), pandemic wave (beta, delta, omicron), vaccinated status (fully or not fully vaccinated), geographic location in the Western Cape (Cape Town vs. other), diabetes, hypertension, chronic kidney disease, prior COPD, and previous tuberculosis.
- The authors estimated that when including all patients who survived at least 2 days after admission, use steroids was associated with a significant reduction in in-hospital mortality (adjusted hazard ratio 0.49, 95% CI 0.30 to 0.86). Estimates of effect were sensitive to this assumption, with more muted benefits the longer we set the cut-off for cohort inclusion—see the table below:

| **Restriction** | **Adjusted Hazard Ratio for Death (95% CI)** |
| --- | --- |
| Must survive to day 2 (base case) | 0.49 (0.30 to 0.79) |
| Must survive to day 3 | 0.56 (0.34 to 0.92) |
| Must survive to day 4 | 0.62 (0.38 to 1.02) |
| Must survive to day 5 | 0.72 (0.42 to 1.19) |

- Beyond the data contained in the main text and Table 3, additional subgroup analyses were performed, using our base case assumption that patients must survive to at least day 2. These data reporting the adjusted hazard ratio for death are in the table below.

| **Outcome** | **Subgroup** | **Adjusted Hazard Ratio (95% CI)** |
| --- | --- | --- |
| Death | Wave 2 Only (Beta Wave) | 0.15 (0.04 to 0.65) |
|  | Wave 3 Only (Delta Wave) | 0.66 (0.36 to 1.20) |
|  | Wave 4 Only (Omicron Wave) | 0.71 (0.25 to 2.02) |
|  | Unvaccinated of Partially Vaccinated Only | 0.47 (0.29 to 0.77) |
|  | Fully Vaccinated Only | Not estimable |

*Note some estimates not possible due to too few events in one group.
