# Supplementary material for: The association of SARS-CoV-2 infection and tuberculosis disease with unfavorable treatment outcomes: A systematic review
Source: PLOS Glob Public Health. 2023 Jul 19;3(7):e0002163. doi: 10.1371/journal.pgph.0002163 (PMC10355446; doi:10.1371/journal.pgph.0002163)
Supplement: S3 Text — (DOCX) [file pgph.0002163.s003.docx]

# S3 Text. Additional Results

*Coinfection Review*

**Risk of Bias**

1. Risk of bias related to selection and group assignment: Overall, risk of bias for categories in this domain was varied between studies. We found a high risk of bias for 7/8 (88%) included studies with respect to the representativeness of the included patient population.^2-8^ This was primarily because they were largely individuals recruited in inpatient settings and therefore may be sicker and/or have characteristics that increase their likelihood for unfavorable outcomes, but also because individuals included were only those with known treatment outcomes, or those with symptoms consistent with COVID-19. There was a low risk of bias for 6/8 (75%) studies in terms of recruitment of control and comparator groups, as patients were generally consecutively recruited or had similar indications for SARS-CoV-2 testing.^1,3-5,7,8^ Most (5/8; 63%) studies had a low risk of bias associated with assignment to SARS-CoV-2 infected vs. SARS-CoV-2 uninfected groups, ^2-5^ however, criteria for SARS-CoV-2 and/or TB testing and ultimate diagnosis were unclear in two studies,^1,7^ and in one study, there was high risk of bias as it was a registry-based analysis with no information on timing of testing (i.e., it is uncertain if persons acquired SARS-CoV-2 after an initial negative test).^8^ There was an unclear risk of bias associated with the outcome being present at enrolment in one study (13%) due to it being a retrospective registry-based design, with uncertainty that the end outcome (mortality) had not occurred prior to TB or SARS-CoV-2 diagnosis.^8^
2. Risk of bias related to comparability of populations: Overall, risk of bias for categories within this domain was high. All 8 studies were at high risk of bias due to confounding.^1-8^ Studies were non-randomized, and no study provided adjusted estimates of effect for the outcome of interest, with generally large differences between patients with vs. without SARS-CoV-2 coinfection. Most (5/8; 63%) studies were at high risk of bias related to similarity in management and care as details were often missing on how patients were clinically managed.^1-3,7,8^
3. Risk of bias related to outcome assessment and/or ascertainment: Overall, there was low risk of bias across categories within this domain due to use of outcomes such as mortality and hospital admission/length of stay, which are rarely subject to misclassification. Most studies (7/8; 88%) had a low risk of bias for outcome ascertainment as TB treatment or mortality outcomes were known; ^1-7^ only one registry-based study in India was deemed at unclear risk of bias due to uncertainty in completeness of death records.^8^ Four (50%) studies were at low risk of bias for follow-up duration (i.e., enough time to ascertain outcomes), however for two (25%),^1-3,6^ follow-up time was unclear,^5,8^ and for another two (25%) follow-up ended at hospital discharge.^4,7^ Two (25%) studies had high risk of bias due to >10% loss to follow-up for TB outcomes.^2,3^

**Primary Outcomes**

Three studies assessed unfavorable TB treatment outcomes among persons diagnosed with SARS-CoV-2 within 28 days of TB treatment initiation (n=17 coinfected; n=93 TB disease only).^2,3,6^ In these studies, unfavorable TB treatment outcomes ranged from 0% to 57% among coinfected participants and 26% to 63% among participants with only TB (OR range: 0.78 to 1.80). Four studies assessed unfavorable TB treatment outcomes among participants diagnosed with SARS-CoV-2 >28 days after TB treatment initiation—unfavorable TB treatment outcomes ranged from 0% to 70% among participants with SARS-CoV-2 coinfection and from 0% to 64% among participants with only TB disease (OR range: 0.41 to 1.37).^1-3,5^ In the lone study assessing unfavorable outcomes among participants who had SARS-CoV-2 >28 days prior to initiating TB treatment, 2/16 (13%) with evidence of previous SARS-CoV-2 had unfavorable outcomes compared to 9/35 (26%) among participants without previous SARS-CoV-2.^6^ Stratified TB outcome data by HIV coinfection was provided in one study conducted in South Africa among participants with rifampicin-resistant TB.^2^ There did not appear to be any difference in outcomes based on HIV coinfection: for people living with HIV, unfavorable TB outcomes occurred in 13/18 (72%) participants with SARS-CoV-2 coinfection and 26/41 (63%) with TB disease only; for people without HIV, the corresponding outcomes were 5/9 (56%) and 8/13 (62%). The single study in pediatrics (n=25 overall) did not have any unfavorable treatment outcomes.^5^ All studies reporting primary outcomes used retrospective cohort designs and the majority of each study took place in 2020 (when wildtype and alpha variants largely dominated and vaccines were unavailable), preventing subgroup analyses on factors of study design, year of study, circulating variants, and SARS-CoV-2 vaccination status.

**Secondary Outcomes**

For all cause mortality: A study in India—that only measured all-cause mortality and did not measure any other unfavorable TB outcomes—found 19% (35/184) of individuals with SARS-CoV-2 and TB died, while 470/5225 (9%) individuals with TB disease alone died.^8^ However, this study had unclear risk of bias for outcome ascertainment due to its design and had high risk of selection and misclassification bias—TB patients without apparent COVID-19 symptoms were not systematically tested and may not have been tested again if the initial test was negative; if 40% of all SARS-CoV-2 infections are asymptomatic or pauci-symptomatic (and therefore not tested according to guidelines) this would significantly reduce the observed difference in mortality rates (based on rates of mortality among persons with unknown COVID-19 status; **S2 Text**).^12,13^

For in-hospital mortality where only persons with coinfection experienced this outcome, serious concerns with the representativeness of the exposure group exist as inclusion criteria were fever and respiratory symptoms and/or pneumonia in one study and hospital admission in the other study,^4,7^ where participants in the coinfected group had higher WHO ordinal severity scores on admission (i.e., were sicker).

*Clinical Management Review*

**Risk of Bias**

1. Risk of bias related to selection and group assignment: Risk of bias varied between studies across categories in this domain. All included studies were at high risk of bias with respect to the representativeness of the included patient population.^4,9-11^ This is primarily because rationale for use of steroids and other immunomodulating treatment was unclear and use of other medications (e.g., hydroxychloroquine) occurred, leaving uncertainty in the representativeness of the patient group; in addition, some patients in one study did not have laboratory-confirmed SARS-CoV-2.^10^ Further, in one study,^11^ immortal time bias was a serious concern as included patients had to survive at least 2 days in the hospital for inclusion and exact timing of steroid initiation was unknown thereafter. There was a low risk of bias for 2/4 (50%) studies for recruitment of control and comparator groups, as patients were generally consecutively recruited or had similar indications for SARS-CoV-2 testing and treatment;^4,9,11^ in one study comprising several treatment centres, this information was unclear^10^ and in one study it was unclear if all participants in the comparator group were eligible for treatment.^11^ Most (3/4; 75%) studies had a high risk of bias associated with assignment to receipt of steroids or immunomodulating treatment vs. no treatment groups, largely because in South Africa (where each of the 3 studies at high risk of bias were performed) steroid use may not be captured in electronic systems if prescribed using “ward stock” (i.e., medications available on hand in the hospital), leading to potential misclassification of patients who did receive steroids but are classified as not receiving such treatment.^4,9,11^ In addition, in one study^11^ estimating hazard ratios, exact timing of steroid initiation was unreliable and so groups were made dichotomously, with no time-varying exposures—this would lead to misclassification bias and potentially attribute survival benefit to steroids, even during periods where none were received. There was unclear or high risk of bias for all studies related to biases associated with the outcome not being present at enrollment.^4,9-11^ Timing of steroid or immunomodulating treatment initiation was unclear in all studies and it is uncertain if outcomes (such as critical COVID-19) occurred prior to initiation of such treatment.^4,9-11^
2. Risk of bias related to comparability of populations: Risk of bias for categories within this domain was high or unclear. All studies were at high risk of bias due to confounding.^4,9-11^ Studies were non-randomized,^4,9-11^ and two studies provided adjusted estimates of effect for the outcome of interest.^9,11^ However, confounding by indication is highly likely for all studies investigating use of COVID-19 treatments in coinfected patients as steroids or immunomodulating treatment are more likely to be used in sicker participants or those with specific indications that may not be captured within each study.^4,9-11^ Most (3/4; 75%) of the studies had unclear risk of bias related to similarity in management and care as the studies used health administrative data across multiple sites and across different SARS-CoV-2 waves, where patient management may differ.^9-11^
3. Risk of bias related to outcome assessment and/or ascertainment: Overall, risk of bias was mixed across categories within this domain. Most studies (3/4; 75%) had a low risk of bias for outcome ascertainment as SARS-CoV-2 outcomes were known;^4,9,11^ one study had unclear risk of bias as SARS-CoV-2 outcomes were not known for all participants.^10^ The SARS-CoV-2 outcomes studied, including mortality or length of stay, are generally at low risk of outcome misclassification. Most (3/4; 75%) studies were at high risk of bias due to follow-up duration as observation of participants ended at hospital discharge and subsequent outcomes are unknown.^4,9,11^ One study had high risk of bias due to >10% loss to follow-up for TB outcomes.^10^

**Secondary Outcomes**

Need for mechanical ventilation was evaluated in two studies.^9,10^ In the study where statistical adjustment was not possible,^10^ those receiving steroids had higher odds of mechanical ventilation, which appeared to be modulated by time between SARS-CoV-2 diagnosis and TB treatment initiation (**Table 3**). However, in *Jassat et al*, where statistical adjustment was possible, trends were similar to in-hospital mortality, with no significant impact of steroid use on need for mechanical ventilation, which was consistent in subgroup analyses.^9^ ICU admission was only evaluated by *Jassat et al*, where there did not appear to be any significant impact of steroid use on ICU admission (**S2 Text**).^9^ Length of hospital stay was assessed in one study,^11^ where participants receiving steroids had a median (IQR) length of stay of 17 days (7 to 44), while the corresponding duration for those not receiving steroids was 9 days (5 to 24), although these differences are likely driven by vastly different mortality rates and that steroids are more likely indicated for those hospitalized for longer durations.
