# Supplementary material for: The association of SARS-CoV-2 infection and tuberculosis disease with unfavorable treatment outcomes: A systematic review
Source: PLOS Glob Public Health. 2023 Jul 19;3(7):e0002163. doi: 10.1371/journal.pgph.0002163 (PMC10355446; doi:10.1371/journal.pgph.0002163)
Supplement: S4 Text — (DOCX) [file pgph.0002163.s004.docx]

# S4 Text. References in Supplemental Files

1. Kilic L, Altin S, Gonenc Ortakoylu M, Kanmaz ZD, Tutar T, Ozkan GZ. Co-infection of COVID-19 and Tuberculosis. *Turk Thorac J* 2022; **23**(1): 58-62.

2. Mohr-Holland E, Daniels J, Douglas-Jones B, et al. A positive COVID-19 test is associated with high mortality in RR-TB-HIV patients. *Int J Tuberc Lung Dis* 2021; **25**(5): 409-12.

3. Stochino C, Villa S, Zucchi P, Parravicini P, Gori A, Raviglione MC. Clinical characteristics of COVID-19 and active tuberculosis co-infection in an Italian reference hospital. *Eur Respir J* 2020; **56**(1).

4. du Bruyn E et al. Communicable and non-communicable co-morbidities and the presentation of COVID-19in an African setting of high HIV-1 and tuberculosis prevalence. *medRxiv* 2021.

5. Gubkina M. F. PIY, Yukhimenko N. V., Sterlikova S. S., Khokhlova Yu. Yu., Amansakhedov R. B. Outbreak of a new coronavirus infection in a children's tuberculosis department. *Tuberculosis and lung diseases* 2020; **98**(10): 6-10.

6. Gomes et al. Anti-SARS-CoV-2 antibodies seroprevalence among patients submitted to 1 treatment for tuberculosis in Rio de Janeiro, Brazil: a cross-sectional study. *medRxiv* 2021.

7. Zulmansyah NG, Agustian D,Gurnida DA;. Association of Chest X-Rays Features with the Length of Stay in Suspected COVID-19 Status. *GMHC* 2021; **9**(2): 165-70.

8. Kumar MS, Surendran D, Manu MS, Rakesh PS, Balakrishnan S. Mortality due to TB-COVID-19 coinfection in India. *Int J Tuberc Lung Dis* 2021; **25**(3): 250-1.

9. Jassat W, Cohen C, Tempia S, et al. Risk factors for COVID-19-related in-hospital mortality in a high HIV and tuberculosis prevalence setting in South Africa: a cohort study. *Lancet HIV* 2021; **8**(9): e554-e67.

10. TB Covid Global Study Group. Tuberculosis and COVID-19 co-infection: description of the global cohort. *Eur Respir J* 2022; **59**(3).

11. Davies MA, Kassanjee R, Rosseau P, et al. Outcomes of laboratory-confirmed SARS-CoV-2 infection in the Omicron-driven fourth wave compared with previous waves in the Western Cape Province, South Africa. *Trop Med Int Health* 2022.

12. Ma Q, Liu J, Liu Q, et al. Global Percentage of Asymptomatic SARS-CoV-2 Infections Among the Tested Population and Individuals With Confirmed COVID-19 Diagnosis: A Systematic Review and Meta-analysis. *JAMA Netw Open* 2021; **4**(12): e2137257.

13. Yanes-Lane M, Winters N, Fregonese F, et al. Proportion of asymptomatic infection among COVID-19 positive persons and their transmission potential: A systematic review and meta-analysis. *PLoS One* 2020; **15**(11): e0241536.
